# Sensors for Context-Aware Smart Healthcare: A Security Perspective

**DOI:** 10.3390/s21206886

**Published:** 2021-10-17

**Authors:** Edgar Batista, M. Angels Moncusi, Pablo López-Aguilar, Antoni Martínez-Ballesté, Agusti Solanas

**Affiliations:** 1 Department of Computer Engineering and Mathematics, Universitat Rovira i Virgili, Av. Països Catalans 26, 43007 Tarragona, Spain; edgar.batista@urv.cat (E.B.); angels.moncusi@urv.cat (M.A.M.); antoni.martinez@urv.cat (A.M.-B.); 2 SIMPPLE S.L., C. Joan Maragall 1A, 43003 Tarragona, Spain; 3 Anti-Phishing Working Group EU, Av. Diagonal 621–629, 08028 Barcelona, Spain; pablo.lopezaguilar@apwg.eu

**Keywords:** smart healthcare, context-aware environments, user-centric sensors, contextual sensors, Internet of Medical Things, wireless body area networks, information security

## Abstract

The advances in the miniaturisation of electronic devices and the deployment of cheaper and faster data networks have propelled environments augmented with contextual and real-time information, such as smart homes and smart cities. These context-aware environments have opened the door to numerous opportunities for providing added-value, accurate and personalised services to citizens. In particular, smart healthcare, regarded as the natural evolution of electronic health and mobile health, contributes to enhance medical services and people’s welfare, while shortening waiting times and decreasing healthcare expenditure. However, the large number, variety and complexity of devices and systems involved in smart health systems involve a number of challenging considerations to be considered, particularly from security and privacy perspectives. To this aim, this article provides a thorough technical review on the deployment of secure smart health services, ranging from the very collection of sensors data (either related to the medical conditions of individuals or to their immediate context), the transmission of these data through wireless communication networks, to the final storage and analysis of such information in the appropriate health information systems. As a result, we provide practitioners with a comprehensive overview of the existing vulnerabilities and solutions in the technical side of smart healthcare.

## 1. Introduction

Smart healthcare (s-health for short) [1] is a paradigm that advocates for the provision of healthcare services through the use of context-aware environments, equipped with complex sensors, infrastructures and communications networks. From sensors and IoT devices to ubiquitous services and decision-making systems, a plethora of information sources provide data able to augment knowledge on patients, their health status and their context, in order to make better decisions, diagnostics and treatments. The technological landscape is, hence, a key enabler of s-health.

During the early 2000s, the synergies between ICTs and medicine and healthcare practice rapidly converged and enabled a key cornerstone in this field: electronic healthcare (e-health) [2]. The e-health paradigm allowed the provision of online medical treatments and disease management, the sharing of electronic health records in a standardised way, and rapid communications between patients and practitioners, among others. Subsequently, with the generalised use of mobile devices (particularly, smartphones), a novel patient-centric highly-personalised healthcare paradigm emerged: mobile healthcare (m-health) [3]. M-health, considered a linchpin of the provision of today’s healthcare services, streamlines communications between patients and practitioners, and enables remote-monitoring and self-monitoring. Today, many mobile devices, namely smartphones, smartwatches and fitness trackers, already incorporate many sensors for health-oriented purposes.

With the steady implementation of ICTs in the healthcare domain, cities have also started equipping their infrastructures with ICTs to face important demographic challenges such as the growth of the world’s population and the increase in life expectancy. As a result of the progressive integration of sensors in our daily lives, homes, buildings and transportation systems, healthcare facilities and cities as a whole are provided with smart and cognitive capabilities able to collect and analyse vast amounts of heterogeneous data under real-time constraints. The ubiquity and communication capabilities of these environments lead to context-awareness, i.e., environments capable of adapting themselves to users’ needs. The meaningful exploitation of user-centric data in combination with contextual data opened the door to smart healthcare services, aiming to acquire advanced high-level knowledge and providing more effective, cost-efficient, personalised and sustainable healthcare models [4,5,6,7]. Unlike previous paradigms, s-health was the first paradigm that considered the contextual perspective, so it is seen as a particular case of the e-health and m-health paradigms.

To properly deploy efficient smart healthcare services, stakeholders must be aware of all the concerns surrounding the management of sensor data, including its collection, storage, transmission, analysis and presentation. Nowadays, there exists an overwhelming number and variety of devices with sensing capabilities, with different features, technologies, complexities, dimensions and costs. These sensing devices, able to collect and transmit data from multiple physical locations, are paramount to enable the contextualisation of smart environments, such as smart homes, smart hospitals and smart cities. More specifically, such devices are topologically organised as networks, mostly wireless to endow the system with major flexibility and cost-effectiveness. In this scope, wireless sensor networks (WSNs) and wireless body area networks (WBANs) emerged and attracted the attention of many stakeholders from very different industries, namely healthcare, sport, entertainment, environmental, transportation or manufacturing, among others [8,9,10,11]. However, a number of challenges could arise from these communications in terms of throughput, latency, reliability, availability and security.

Concerning the latter, data security stands as one of the most important features in the healthcare domain. Medical data, such as electronic health records, biomedical signals and physiological parameters, are highly sensitive and must be handled with the highest security and privacy standards. Moreover, the high value that such data might generate in the black market motivates attackers to infiltrate themselves into the information systems [12,13]. Despite security safeguards, the history of both communication networks as well as that of the Internet encompasses countless security flaws, vulnerable cryptographic protocols and threatening data breaches. Every information system or communication network is hence virtually prone to be attacked by cybernetic criminals or suffer from irreparable damages because of unintentional human errors. Smart health systems, involving a large number of complex and heterogeneous devices and entities, may present risks from a security and privacy perspective unless properly considered.

The possibilities of s-health applications are many and varied. Hence, facing of all them individually is a daunting task since the use of specific sensors, devices and technologies highly depends on the particular requirements of each application and service. In consequence, the security threats and countermeasures can vary among them. To help readers properly understand all the challenges involved at the time of developing any s-health application, this article adopts a more neutral and high-level approach, by focusing more on the individual actors involved in these scenarios, rather than the very applications. Notwithstanding, numerous examples have been provided to contextualise the topics addressed to the smart health domain. All things considered, it is essential to provide a comprehensive approach to the realistic deployment of smart healthcare services. To this end, the following aspects must be considered:

- The complete variety of attributes related to people’s health status and their context must be considered so that the s-health service is as beneficial as possible. Sensing devices must be properly selected in accordance with their inner requirements such as accuracy, reliability, dimensions, computational capabilities, cost or power consumption, among others.
- Proper communication technologies must be favoured according to the specifications of the sensing devices and considering the complex nature of WSNs and WBANs. Scalability, density, coverage area and security are important aspects to be taken in mind for communications.
- All s-health-related ICT components must meet with the highest security requirements to thwart malicious activities. All in all, information security and data privacy, as well as the adequate protection of devices, networks and services, is first and foremost.

There is plenty of literature related to IoT, WBANs and their security concerns. The article in [14] reviewed the most recent sensing techniques for monitoring health conditions, including flexible electronics and textile-based wearables. Similarly, the work in [15] also analysed wearable, non-invasive sensors to monitor vital signs. From a more practical approach, the authors in [16] provided a comprehensive review on the benefits and opportunities of using IoT and wearables in health-oriented applications. In [17], the authors presented a comprehensive survey and classification of commercially available wearables and research prototypes. The articles in [18,19,20] presented the most common technologies for WBANs and contextualised their applicability in the health domain. In [17,21], the authors presented a comprehensive survey of the attacks and countermeasures to WBANs.

Inspired by this research, this article contextualises all these elements into the smart healthcare paradigm and provides a more comprehensive approach within this domain considering the data life-cycle, from its collection, going through its transmission, to its final exploitation. Often forgotten, this article also surveys the sensors and security concerns related to contextual sensing, a required dimension in smart health. To the best of our knowledge, this is the first article to present a concise and comprehensive review of all the aforementioned smart healthcare research. Therefore, with the aim of contributing to the proper deployment of secure smart health systems, this article provides a down-to-earth landscape of the sensors and communication technologies that could be used to enable these scenarios. More specifically, the contribution of this article is three-fold. First, we provide a detailed review and characterisation of sensors, either user-centric or contextual, that enable smart healthcare services. Second, we discuss the most common wireless communication technologies that allow those sensors to interoperate and transfer the sensed information in a secure manner. Furthermore, third, we also elaborate on the most serious vulnerabilities and threats in such settings, and suggest the corresponding countermeasures. All in all, we hope that this article helps technicians, practitioners, stakeholders and researchers to set the grounds for more secure and private context-aware environments and smart healthcare services.

The rest of the article is organised as follows. Section 2 provides a thorough overview of the sensors used for gathering user and context attributes that are of interest for smart health purposes. Then, Section 3 describes a three-tier WBAN architecture for deploying complex s-health services, and summarises the most relevant wireless communication technologies enabling this architecture. Then, Section 4 analyses the main security aspects associated with smart healthcare environments by discussing the main threats, vulnerabilities and possible countermeasures. Considering the previous observations, Section 5 provides an extensive discussion on open issues and research opportunities to be addressed in the future. Finally, the article closes in Section 6 with some final remarks.

## 2. Sensors: Definition and Taxonomy

Advancements in microelectronics and manufacturing technologies have enabled the development of a large variety of sensors, embedded in electronic small-scale devices, with high sensitivity, low energy consumption and contained costs. This section elaborates on the different sensors to be considered within the s-health paradigm. Sensors are categorised into two groups according to the nature of the sensor data: user-centric data, i.e., referring to personally identifiable individuals; and contextual data, i.e., referring to the context or the immediate environment. Table 1 summarises all the sensor data described in this section.

### 2.1. User-Centric Sensors

**Figure 1:**
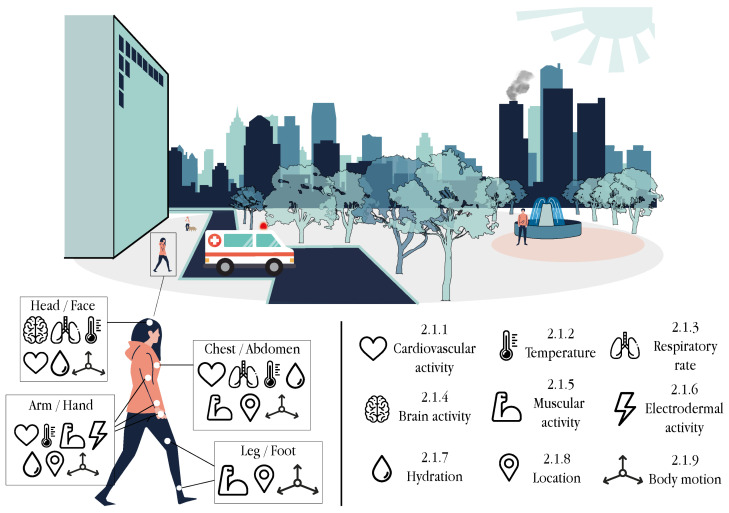
User-centric attributes for smart healthcare: each icon, representing the sensors of an attribute, is assigned to a part of the body where that attribute can be collected.

User-centric sensors aim to acquire specific data related to the individuals themselves. Within this context, most of these data relate to medical data such as biosignals (i.e., physiological parameters), health status and health conditions. Moreover, sensors collecting the location or the body movements of individuals are also of interest to contextualise individuals. All these sensors have already been seamlessly integrated within wearable devices, whose popularity has grown during the last decade as part of the IoMT technology [14,15,22]. In particular, wearable technology has revolutionised ubiquitous computing with low-cost yet powerful devices, including body-worn accessories, smart textiles, garments, on-skin tattoos, ingestible sensors and implantable appliances, among others [17,23]. This technology, capable of monitoring, analysing and transmitting individuals’ data, opens the door to numerous healthcare opportunities, ranging from the remote or self-monitoring of patients’ health to the early detection of medical complications. All in all, the generalised use of wearables is contributing to reducing healthcare expenditure and shortening medical times, which leads to more sustainable healthcare models. This section reviews some of the most common methods for the sensing of user-centric attributes (Figure 1) which are summarised in Table 2, Table 3, Table 4, Table 5, Table 6 and Table 7.

#### 2.1.1. Cardiovascular Activity

Cardiovascular diseases are the first cause of mortality worldwide, responsible for one third of all global deaths [24]. Therefore, the gathering of cardiovascular parameters—namely heart rate, blood pressure, oxygen saturation and blood glucose concentration—has become commonplace in many wearable and IoMT devices [25] (see Table 2). The continuous monitoring of these parameters contributes to cardiac rehabilitation recovery [26] and to early detect abnormal conditions (e.g., tachy/bradycardia, hyper/hypotension, hyper/hypoglycemia…) that might lead to cardiovascular emergencies, such as arrhythmia, stroke or even death [27,28].

One of the most commonly measured vital signs is the heart rate (or pulse), i.e., the number of heartbeats per minute. Electrocardiography (ECG), photoplethysmography (PPG), ballistocardiography (BCG) and phonocardiography (PCG) are common methods of heart rate sensing [29]. On the one hand, ECG sensors record the electrical activity and rhythm of the heart, in the form of electrocardiograms, by attaching a series of electrodes to the skin. Traditional ECG monitoring uses Holter monitors, well-known medical portable units, able to capture long recording periods (from days to weeks) in both home and hospital environments. Despite the high reliability of these devices, they are quite obstructive, invasive and uncomfortable due to their wired architecture. To overcome this, wireless ECG monitoring solutions integrated in wearable devices have already been proposed [30,31,32,33]. Alternatively, heart rate data can also be acquired using optical PPG methods by means of pulse oximeter sensors, which measure the intensity of an LED light reflected or transmitted through the skin affected by the changes in the blood volume during each heartbeat. Due to the low-cost and non-invasive nature of this method, most wearables, fitness trackers and commercial devices opt for PPG methods [34,35,36]. However, the accuracy of PPG measurements is strongly affected by several factors including the measurement procedure, environmental factors and skin pigmentation, among others, and post-processing techniques are desirable to enhance quality [37,38,39]. Finally, body motion-based BCG methods [40] and sound-based PCG methods [41] are less prominent in wearables. Monitoring a heart’s rhythm is particularly important in at-risk patients who may sometimes require an immediate response in a proactive fashion. Hence, actuators are likely to play a key role in the years to come. Today, wearable, non-invasive and non-implantable cardioverter-defibrillators are already a reality [42,43,44].

Another popular measurement is the blood oxygen level, which indicates how well the oxygen is distributed to every cell, tissue and organ. Monitoring this attribute is fundamental for people suffering from blood disorders (e.g., anaemia), circulatory problems or respiratory diseases (e.g., asthma and COPD) in order to prevent shortness of breath, hypoxia or cyanosis. The most accurate method to measure the blood oxygen level is the arterial blood gas (ABG) test which analyses blood samples using specific analyser devices. However, this procedure is invasive and painful and it is typically conducted in hospital settings only. More aligned with the smart healthcare paradigm is the use of PPG-based oximeter sensors, already used for heart rate sensing, to measure the blood oxygen saturation (${\mathrm{SpO}}_{2}$). Compared to ABG tests, this method is simpler, cheaper, non-invasive and non-painful, but slightly less accurate [45,46]. Many wearables have been designed to monitor this attribute from different parts of the body, such as the wrist, ear and finger [47,48,49,50], and even using smart textiles [51].

Cardiovascular activity monitoring also considers blood pressure, i.e., the force with which the blood moves through the circulatory system. High blood pressure, called hypertension, is a serious cardiovascular risk with no warning symptoms (popularly known as a “silent killer”) that must be fought with healthy lifestyles. Cuff-based sphygmomanometers are the traditional in-hospital devices to measure this attribute. Although they are cheap, accurate and even available in portable electronic devices for home monitoring, they are invasive and unsuitable for outdoor usage, long-term monitoring no real-time monitoring. To overcome this, sensing technologies have contributed to developing non-mechanical and non-invasive solutions [52]. Among others, accurate blood pressure results can be estimated from the pulse transit time (PTT) measure obtained from the combination of PPG and ECG signals [53,54,55]. Many devices, including smartphones, smartwatches, bands and patches have been designed to this aim [56,57,58,59].

Finally, blood glucose concentration is crucial for the management of diabetes. Unless properly managed, diabetic people may experience severe or tragic consequences. Therefore, many m-health applications have emerged with the aim to monitor, suggest and engage diabetic people with their treatment [60,61,62]. Traditionally, blood glucose is measured using glucose meters, portable devices with electrochemical sensors that chemically analyse a blood drop, typically obtained from the fingertip. Although these devices are easy to use, cheap and accurate, they are invasive and do not provide continuous monitoring. For an s-health oriented approach, several electrochemical-based sensor solutions were implemented in wearable patches, tattoos and implantable sensors [63,64,65]. Optical-based measurements, already used to measure other attributes, have also been proven for blood glucose monitoring through spectroscopy techniques and infrared technology. Although results are less accurate, solutions are far less invasive and obstructive [66,67]. Complementary to these sensors, diabetes management can reach a higher dimension, by actively monitoring blood glucose in diabetic patients and deliver, when necessary, insulin through in-body insulin pump actuators [68].

#### 2.1.2. Temperature

The body temperature (or core temperature) is an indicator of the overall physiological status of an individual and helps determine illnesses such as hypothermia, fever, heat stroke or circulatory shock, among others. Unfortunately, standard medical measurements using thermometers are invasive, obstructive and not aligned with s-health solutions. Nevertheless, these measurements can be estimated in a less-invasive way from one’s skin temperature, i.e., the temperature of the outermost surface of the body, generally obtained from the wrist, arm, armpit, chest or forehead [69] (see Table 3).

Thermistors, thermocouples and optical methods are common means of measuring skin temperature [70]. In particular, thermistor sensors are generally popular, cost-efficient and ideal for many wearables, yet their accuracy is influenced by a number of factors including the measurement’s location (e.g., wrist, forehead, etc.), ambient temperature (e.g., hot or cold environment), sensor strain, sweating and the distance between the sensor and skin [71,72,73,74,75]. Optical methods, based on FBG or infrared technology, are quite similar to thermistors in terms of accuracy, comfortability and cost [76]. On the other hand, thermocouples’ sensors, despite also being cost-efficient, have worse accuracy than the previous methods. Although many sensors have been integrated in traditional wearables, further non-intrusive and more comfortable solutions for temperature sensing have already been proposed by means of stretchable and flexible patches [77,78,79] and smart textiles [51,76,80,81].

#### 2.1.3. Respiratory Rate

The number of breathing cycles per minute determines the respiratory rate, one of the main vital signs of the human body and a clear indicator of overall health. Monitoring this attribute helps identify or keep track of disorders, such as asthma attacks, panic attacks, sleep apnoea, shortness of breath, COPD, pneumonia or anaemia. Although variations can be caused due to the age or the physical activity, abrupt or prolonged abnormal respiration rates can lead to permanent injury or death [82]. In contrast to other vital signs, the recording of one’s respiratory rate is less automated (see Table 4). The gold standard technique for its measurement consists of counting the number of times that one’s chest or abdomen rises during one minute while the patient is resting. This manual technique is insufficient for smart health.

More automated, the most popular monitoring methods are contact-based, i.e., the sensor directly contacts the skin [83]. Breathing can be monitored considering the expansion and contraction movements of the chest walls using strain sensors (e.g., resistive, capacitive and inductive sensors) [84,85], transthoracic impedance sensors [86,87,88], or motion sensors (e.g., IMUs, later introduced in Section 2.1.9) [89,90]. In general, this technique provides notable accuracy (even though body motion artefacts and environmental factors can diminish the quality) and the small dimensions, low power consumption and contained costs of these sensors facilitate their integration into wearable devices and textiles to be minimally intrusive [91,92,93,94].

Further contact-based methods exist for respiratory rate sensing, although they might be less accurate or more obstructive in smart health. For instance, acoustic methods, which aim to characterise the respiratory sounds using microphones [95,96,97], are promising and suitable in wearable devices, however, unfortunately, they are extremely susceptible to environmental noise, so they should only be considered under very controlled scenarios. Furthermore, one’s respiratory rate can also be measured according to the temperature difference between the inhaled air and the exhaled air, by means of electric-based temperature sensors such as thermistors, thermocouples and pyroelectric sensors, placed close to the nose or the mouth [98]. However, this method is quite obstructive and also extremely sensitive to environmental factors. Similarly, inhaled and exhaled air can also be compared in terms of humidity. In this context, electric-based sensors—such as capacitive and resistive sensors—are common, although solutions based on nanoparticles and fibre-optic technology are gaining increasing importance [99]. However, as before, intrusiveness and sensitivity to external factors make this method only suitable for controlled scenarios. Last, there is a growing interest in modulating the cardiac activity recorded from ECG and PPG signals to measure the respiratory rate [100,101,102,103]. The lowly invasive, low energy consumption and reduced cost of this method have fostered research in this direction to overcome the main limitations of the aforementioned methods.

Another group of monitoring methods are contactless, i.e., the sensor does not directly contact the skin, which are more comfortable solutions which facilitate long-term monitoring and monitoring during sleep [104]. The main drawback of such methods is their noise sensitivity to environmental or motion artefacts, so they should only be considered under very constrained environments. Most contactless methods are based on camera sensing, which estimate the respiration rate by tracking one’s chest movements [105,106]. Other methods have proposed infrared thermal imaging sensors to detect the temperature fluctuations during the respiration cycle [107] or ultrasonic proximity sensors [108,109]:

#### 2.1.4. Brain Activity

Neurological disorders are one of the most prevalent disorders in our society, including Alzheimer’s disease or other forms of dementia, epilepsy, meningitis, traumatic brain injury and cerebral palsy. Unfortunately, classical brain activity monitoring methods are highly sophisticated and require large and expensive instrumentation. Hence, developing novel methods considering the size, cost and power constraints so as to be integrated into portable and wearable devices is truly challenging (see Table 5).

One of the most popular methods for assessing the quality of brain activity is electroencephalography (EEG) which measures the electrical activity in the brain by placing small electrodes at multiple locations on the scalp. Conventional EEG measurements, conducted in medical facilities, require a head cap with electrodes connected to a recording instrumentation through long wires. Although accurate, this method is significantly obstructive and invasive. To face these shortcomings, wireless technologies have enabled comfortable EEG monitoring using wearable devices [110,111]. Headset-based solutions are the most prominent [112,113,114], although there less-obstructive wearables also exist which are placed on the forehead [112,115] or the ear [116,117]—even more discreet solutions include temporary tattoos [118]. In addition to EEG, other methods for brain activity monitoring, albeit less frequently integrated in wearable devices due to their complexity, are based on functional near-infrared spectroscopy (fNIRS) for hemodynamic changes [119,120], magnetoencephalography (MEG) considering magnetic fields [121] and positron-emission tomography (PET) [122].

#### 2.1.5. Muscular Activity

Monitoring muscular activity can help detect and evaluate the severity of neurodegenerative diseases such as Parkinson’s disease, bradykinesia or dyskinesia symptoms [123,124]. Non-invasive wearable sensor technology can be beneficial in the early detection of these disorders in non-diagnosed patients and to remotely monitor the evolution of these conditions in already-diagnosed patients (see Table 5).

The most popular diagnostic procedure to assess the functioning of the muscles and the nerve cells is electromyography (EMG) which measures the electrical signals generated by the muscles during their movement. There are two kind of methods for EMG recordings. On the one hand, intramuscular EMG methods are invasive, potentially painful, and not well aligned with smart healthcare solutions. On the other hand, surface EMG methods are non-invasive procedures that only require placing some patch electrodes on the muscle’s skin, facilitating their integration in wearable devices, such as wristbands, armbands, caps or even textiles, to enable long-term monitoring in real-time [125,126,127,128], tracking tremor and dyskinesia symptoms [129], preventing falls [130], recognising gestures and activities [131], controlling robotic prosthetics [132,133,134] and rehabilitation [135,136]. Although more comfortable, the quality of these measurements is affected by the skin’s properties, tissue structure, the adherence of the electrodes to the skin and external electromagnetic interference and noise-filtering techniques are required [137].

In addition to electrical measurements, muscular activity can also be measured from a mechanical perspective through mechanomyography (MMG) [138], which measures the mechanical vibrations of muscles’ fibres using accelerometers, condenser microphones, piezoelectric pressure sensors or force-sensitive resistors [139]. This method has been widely used to recognise gestures useful for the control of robot prosthetics or for rehabilitation [139,140,141], as well as to assess muscles conditions [142].

#### 2.1.6. Electrodermal Activity

Electrodermal activity (EDA), also known as skin conductance or galvanic skin response, aims to detect changes in the electrical properties of the skin, especially due to sweating. This property, highly valuable in behavioural medicine, allows detecting emotional states, such as stress, anxiety, depression, fatigue or risk [143,144,145], characterise sleep activity [146,147] and manage the neurological status [148,149].

The instrumentation required to measure EDA is simple and only requires a couple of electrodes placed next to each other on the skin surface, generally the wrist or fingertip (see Table 6). Different types and materials of electrodes are currently being investigated in order to be integrated into wearable devices, considering both signal quality and comfort aspects for long-term monitoring [150]. Although initial devices were wired, many current solutions, based on wristbands [145,151,152] and finger straps [144,153] are already wireless.

#### 2.1.7. Hydration

Hydration plays a significant role in people’s health status. Hot environments or strenuous physical exercise (e.g., high-performing athletes or military training recruits) can accelerate the appearance of dehydration, a dangerous condition that leads to physical and cognitive performance loss. Furthermore, monitoring the (de)hydration level in elderly people, a very high-risk group, is crucial to prevent their fragile health condition worsening. Prolonged dehydration periods can lead to serious diseases, such as kidney disease, heart diseases or respiratory infections [154,155].

Traditionally, the method to assess dehydration is qualitative, i.e., looking directly at the patient’s eyes or lips. To measure the hydration level in a quantitative way, several techniques based on optical spectroscopic, electromagnetic or electrochemical measurements have been proposed [156,157,158,159] (see Table 6). Hydration sensors are commonly integrated in stretchable epidermal sensors [156,157,160] and wristbands [155,158], but they have also been successfully designed as patches and headbands [155,158,161], and even integrated in smart textiles [162].

#### 2.1.8. Location

The provision of health services is not restricted to healthcare facilities only. Checking one’s health status or collecting physiological parameters either at home or during walks/exercise by means of wearables is commonplace nowadays. Therefore, other non-medical user-related data are a valuable complement to medical data so as to contextualise users. In particular, thanks to the self-location capabilities integrated in most smartphones and smartwatches, location data are extremely valuable to assist at-risk people, including the elderly, children and people with some certain conditions (see Table 7). More specifically, healthcare-oriented LBS can contribute to finding medical assistance nearby, notify the emergency services of the exact location of the emergency, prevent disorientation and wandering episodes, and even provide walking recommendations to foster healthier lifestyles [5,163,164].

People’s location is usually determined using GPS, integrated in most smartphones, wearables and IoT devices. This satellite-based technology is highly accurate for deploying smart health services in outdoor environments—although its accuracy deteriorates indoors or under bad weather conditions. Other satellite-based positioning solutions include GLONASS and Galileo, but their availability in mobile device is less popular. Regarding indoor locating, a number of different technologies have been proposed for precise indoor positioning and proximity-based systems, such as Bluetooth Low Energy (BLE) beacons [165], WiFi-based positioning system (WPS) [166], radio frequency identification (RFID) [167] and ultra-wideband (UWB) [168] technologies (further details can be found in Section 3.2). These technologies help locate and keep track of the people’s trajectories in indoor environments, such as the elderly in nursing homes or patients in a smart hospital.

#### 2.1.9. Body Motion

Human motion analysis helps physicians and physiotherapists identify abnormal movements and plan and assess the correctness of rehabilitation programmes [169] (see Table 7). More specifically, applications of body motion measurements in healthcare are diverse, including the analysis of gait patterns and the assessment of gait abnormalities [170,171,172]; the development of corrective posture systems for rehabilitation purposes; enhance athletes’ performance [173,174,175]; the detection of falls (especially for the elderly) [176,177,178]; and the recognition of gestures and activities [179,180].

Many solutions are based on optical motion capture methods, aiming to track human motions in a 3D space using multiple cameras triangulating markers attached to different parts of the body. Although being largely used in computer-generated special effects in cinema and TV and their high potential, these systems are very complex and require expensive time-consuming operations. Moreover, their use in indoor settings is only feasible under very controlled environments [181,182]. There are alternative optical methods that use RGB-depth cameras that do not require the attachment of markers to the body. Although these methods are more practical and less invasive, they do not provide spatio-temporal information (i.e., there is no way to know whether the gesture is beginning or finishing) and they are also only viable under controlled environments [183,184,185]. Hence, capturing body motion and human movements in real-life, uncontrolled environments is not straightforward. The use of inertial measurement units (IMUs), which considers a triaxial accelerometer, a triaxial gyroscope and a non-inertial triaxial magnetometer, enable the recreation of the motion of the movements [186]. This method, which works in both indoors and outdoors, can be integrated in wearable devices for enhancing people’s comfort while enabling long-term monitoring [170,187,188].

### 2.2. Contextual Sensors

**Figure 2:**
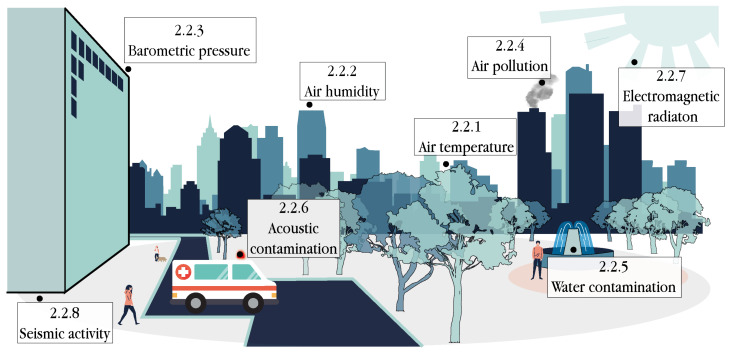
Contextual attributes for smart healthcare that can be sensed from context-aware environments.

The deployment of massive networks of IoT devices collecting contextual parameters in real-time has enabled context-aware environments, where smart health applications can be deployed. Whereas this information is rarely used in classical healthcare paradigms, it plays a key role in the s-health paradigm to provide more efficient and effective health services and improve people’s health status and welfare. This section reviews the most popular methods for the sensing of contextual attributes (see Figure 2), which are summarised in Table 8, Table 9 and Table 10.

#### 2.2.1. Air Temperature

Air temperature is one of the main contextual parameters contributing to the comfort, welfare and health of people. Extreme temperatures or high temperature variations can have negative consequences for human health. Especially important with the incoming climate change effects, several studies have aimed to establish a relationship between air temperature and the mortality rate [189,190,191,192] or the appearance of diseases and disorders [193,194,195,196].

There are different types of temperature sensors integrated in a large number and variety of IoT devices [197] (as can be seen in Table 8). Thermocouples are popular sensing solutions due to their contained costs, rapid responses to temperature changes and large range of temperature detection values. However, accuracy is the main drawback of this kind of sensors, and they should not be used when very precise measurements are needed.

Resistance temperature detectors, with a metal core, are more accurate, but their response to temperature changes is slower and their cost is higher compared to other sensors. Similarly, thermistors are low-cost sensors with a ceramic or polymer core that lead to faster responses to temperature changes with notable accuracy. Last, several devices prefer semiconductor-based sensors using integrated circuits because of their low cost, low energy consumption and fair accuracy.

#### 2.2.2. Air Humidity

Abnormal humidity values can cause physical discomfort and lead to serious health outcomes [198]. On the one hand, low humidity can cause nasal congestion which increases the risk of flu and other respiratory infections [199,200]. On the other hand, high humidity together with high temperatures can lead to hyperthermia, dehydration, heat exhaustion and heat stroke. Interestingly enough, high humidity also fosters the appearance, growth and spread of bacteria and viruses that can aggravate health conditions, including infectious diseases, allergies and respiratory problems, among others [200,201].

Similarly to air temperature sensing, many IoT devices integrated mechanisms for air humidity monitoring, which can be measured using different types of sensors (see Table 8). Capacitive humidity sensors are commonly used in the market due to their accuracy (although it decreases in extreme environments), small dimensions, low power consumption and wide measurement range. Furthermore, resistive sensors were inexpensive and suitable solutions in non-extreme environments that do not require extremely precise results [202]. More recently, optical methods based on fibre-optic sensors offer several advantages regarding the aforementioned sensors in terms of durability, higher accuracy, reduced temperature dependency and electromagnetic immunity [203,204]. Although this method is less used in commercial devices, its future is encouraging.

#### 2.2.3. Barometric Pressure

Differences in barometric pressure because of weather shifts or altitude changes may harmfully affect the human body. Headache, migraine attacks or joint pain, such as arthritis, are common symptoms appearing due to pressure changes [205,206,207,208]. Current barometric pressure sensors are based on microelectromechanical systems (MEMS), based on the piezo-resistive effect, which offer high accuracy, low power consumption, low cost and can be manufactured at low cost and small dimensions so that they could be seamlessly integrated into portable IoT devices [209,210,211] (see Table 8).

#### 2.2.4. Air Pollution

Air pollution is a global public health emergency: nine out of ten people breath air containing high levels of pollutants, resulting in the death of approximately seven million people worldwide annually [212]. The most harmful pollutants include particulate matter (P$M_{10}$, P$M_{2.5}$ and P$M_{0.1}$), ozone ($O_{3}$), carbon monoxide (CO), nitrogen oxide (N$O_{2}$), sulphur dioxide (S$O_{2}$), lead, polycyclic aromatic hydrocarbons (PAHs), volatile organic compounds (VOCs) and dioxins, which are more concentrated in large urban areas or industrialised regions. These pollutants contribute to respiratory problems, such as COPD, asthma and bronchitis, cardiovascular diseases, neurological disorders, reproductive dysfunctions, skin diseases and a variety of cancers in long-term exposures [213,214,215,216]. Smart health applications can intelligently manage this information to reduce the exposure of citizens to pollutants.

Optical spectroscopy methods are standard analytical techniques to detect gas pollutants in the air, although they are time-consuming, expensive and cannot be used in real-time. More interestingly, for smart health, there are two main low-cost sensing methods for measuring this information more efficiently [217,218] (see Table 9). Commonly used in the industry are metal oxide semiconductor (MOS) sensors, characterised by their low cost, small dimensions, fast response times, low power consumption and high durability. However, they are sensitive to changes in environmental conditions and to interfering gases. Such limitations are overcome using electrochemical sensors, even though their cost and dimension are significantly higher in comparison to MOS sensors [219,220,221].

#### 2.2.5. Water Contamination

In addition to air, water is another vital resource that may contain microbiological or chemical contamination. Poor quality water, especially abundant in low-income countries, can lead to waterborne parasitic infections (e.g., cholera, dysentery and typhoid), chronic diseases, reproductive complications and adverse neurodevelopment procedures [222].

Traditional methods for measuring water quality are based on laboratory chemical analyses of water samples, which are manually collected at various locations and at different time periods. Despite being accurate, this procedure is inefficient, resource-consuming and offline because no real-time information is provided—which is essential to detect outbreaks of contaminated water (see Table 9). To evaluate the quality of water in real-time, electrochemical sensors can monitor changes in water parameters that become affected by chemical and biological pollutants, such as turbidity, free/total chlorine, oxidation-reduction potential, electrical conductivity, pH, nitrates level and temperature [223,224,225]. Furthermore, further approaches have proposed the detection of floating debris in contaminated water by means of aquatic sensors embedding a CMOS camera [226].

#### 2.2.6. Acoustic Contamination

Regular exposure to acoustic contamination, this is, elevated sound levels and environmental noise, can result in adverse health outcomes, including hearing impairments, sleep disturbance, chronic stress and an increased incidence of suffering cardiovascular and metabolic diseases [227,228,229]. Today, the continuous and real-time monitoring of noise levels is possible using low-cost and small microphones embedded in IoT devices [230,231] (as can be seen in Table 9).

#### 2.2.7. Electromagnetic Radiation

Electromagnetic radiation has become a popular form of pollution due to the omnipresent telecommunication equipment. In short, two types of radiation exist: non-ionising radiation and ionising radiation. On the one hand, ionising radiation (e.g., Gamma rays, X-rays and higher UV light band) can cause tissue damage since rays contain sufficient electromagnetic energy to detach electrons from atoms or molecules. Numerous studies state the adverse health outcomes due to prolonged exposures to ionising radiation: UV exposures can lead to sunburns, eye damage and skin cancer, X-rays can modify cells’ genetic material and cause mutations and cancer and Gamma rays can cause disorders, such as leukaemia and bone, breast and lung cancer. Moreover, syndromes related to the nervous system and neuropsychiatric-related problems, including insomnia, chronic fatigue, sexual dysfunction and memory problems, have also been associated with electromagnetic radiation [232,233,234]. On the other hand, non-ionising radiation (e.g., radio-frequency, microwaves, infrared, visible light and low UV band) may generate thermal energy and excite molecules, but it does not contain sufficient energy to remove electrons from atoms or molecules [233], although concerns have also been raised about its impact on human health [235].

To protect against radiation exposure, portable and affordable devices are being developed to detect Gamma radiation using Geiger–Müller tubes or fibre-optic radiation sensors [236,237,238,239,240] and to detect infrared and UV lights through optical sensors as well [241] (see Table 10).

#### 2.2.8. Seismic Activity

Tremors on the Earth’s surface involve seismic activity resulting from natural disasters, such as earthquakes, volcanic eruptions and explosions. Surface vibrations are a common daily phenomenon, but they are imperceptible for humans due to their low intensity and do not suppose an apparent risk. However, sudden high-intensity shakes can produce seismic waves able to collapse buildings and trigger catastrophic consequences for people, including death. Continuous seismic monitoring does not contribute to people’s health status per se under normal conditions, but it can be essential for alerting or predicting seismic events so to guarantee people’s safety.

Traditional sensing solutions use seismometers, which are reliable and high-performance instruments though nonetheless bulky, expensive and sensitive to electromagnetic interference (see Table 10). In recent years, this activity has being monitored using the inexpensive tri-axial accelerometer sensors that, in combination with machine learning techniques, allows detecting or predicting seismic events [242,243,244]. Additionally, opto-mechanical sensors based on optical fibre technology have already been assessed to monitor ground motions [245].

## 3. Communication Architecture and Technologies

Smart healthcare services are not fed from a single sensing device, but from many of them. To ease this management, devices are logically structured as networks, generally wireless. In this scope, WSNs provide a contextualisation-enabling infrastructure within physical environments for real-time applications. For instance, the coverage of a region with a WSN composed of multiple temperature, humidity and air pollution sensors enables the transmission of real-time data to a smart health service aiming to alert nearby patients with respiratory diseases. Despite the huge potential of WSNs as a whole, it is worth mentioning that each single sensor was generally resource constrained in terms of computation, memory, storage and battery capacity. To increase the life expectancy of the sensors and prevent rapid battery depletion, their power consumption must be as low as possible and the implementation of lightweight protocols is a must—this is particularly the case for data transmission, the most energy-consuming task [246,247].

With the rise of wearable technology, WSNs evolved towards a more user-centric approach: WBANs. WBANs are designed to collect user-centric attributes such as physiological parameters, location and motions, and communicate them to external entities to provide efficient, personalised and real-time health services. Furthermore, actuators within WBANs can receive feedback or commands from other devices and act accordingly. For instance, a diabetes-oriented WBAN enables continuous blood glucose monitoring, and when abnormal values are detected, communicates to an external smart healthcare service and/or activates an insulin pump actuator that delivers insulin into the body. WBANs offer enhanced opportunities concerning active patient monitoring, biofeedback, telemedicine and rehabilitation [18] that can irreversibly shift traditional healthcare models. However, in addition to the technical challenges inherited from WSNs (e.g., latency, throughput, energy consumption…), WBANs have to face additional obstacles for their practical adoption, including reliability, accuracy, fault tolerance, interoperability and security, among others [9,19,248]. The non-compliance of these additional requirements can certainly endanger people’s health. For example, incorrect medical decisions could be taken in the case of the inaccurate sensing of physiological parameters, or transmitting the information through an insecure communication channel.

### 3.1. WBAN Communication Architecture

**Figure 3:**
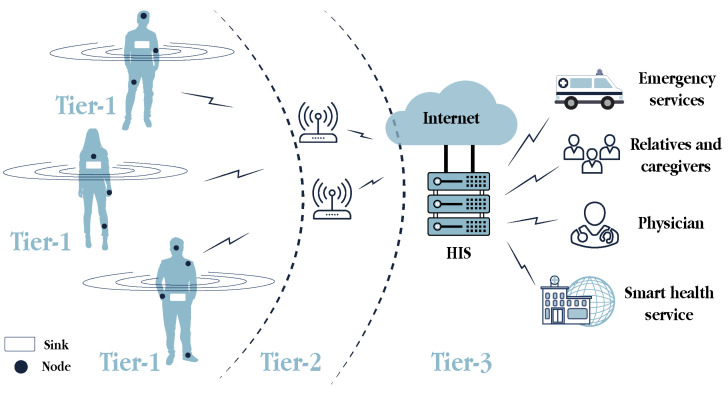
Component-based representation of the 3-tier communication architecture for WBANs.

The low-power and resource-constrained devices involved in WBANs require communication architectures to transmit data in a time- and energy-efficient manner. Fulfilling all of these conditions is one of the most prominent communication architectures for WBAN, which is based on three tiers [19] (as can be seen in Figure 3):

- Intra-WBAN communications (Tier 1): This tier enables communications between the sensors and actuators (i.e., nodes) placed in, on and around the human body, in a range of approximately two meters. In addition to the direct communications among these nodes, they can also communicate with a sink, a portable device attached to the body, to transmit the user-centric data. The sink, which usually refers to a smartphone in the s-health context, is the WBAN coordinator and gateway to the next tier. Short-range and low-energy communication technologies are desirable in this tier.
- Inter-WBAN communications (Tier 2): This tier aims to connect the users’ WBANs with external networks that are easily accessible for other users, such as the Internet and cellular networks. Hence, the communications in this tier take place between the sink and one or more access points, which are gateways to those networks. Large-range communication technologies, such as ZigBee, BLE, Wi-Fi and cellular, were adopted in this tier.
- Beyond-WBAN communications (Tier 3): The communications in this tier refer to those from the health provider. Having received the user-centric data from the previous tier, it was stored in the healthcare information system (HIS) and then, analysed by physicians, medical staff or automatised systems may act accordingly. With the medical records and the profiles of patients, smart healthcare systems can automate real-time diagnosis, adjust medical treatments or alert the emergency services, relatives and caregivers if needed.

**Figure 4:**
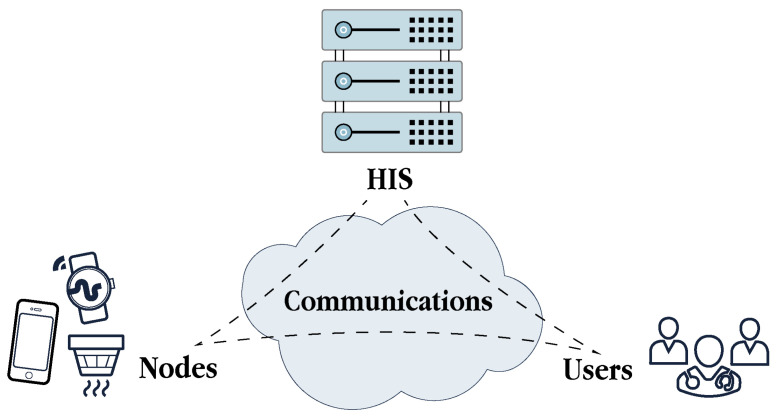
Relationship among the actors involved in smart healthcare systems.

Conceptually, four main actors participate in this architecture (see Figure 4). First, as reviewed in Section 2, nodes are a primary information source in s-health systems. Second, these systems are supported by the HIS, responsible for the storage, retrieval, analysis and presentation of all the data in accordance with the services provided. Third, users intended to use the s-health services, either patients and physicians and must be also considered as crucial actors. Furthermore, fourth, all these actors are able to interact among them thanks to the deployment of communications networks, whose technologies are described as follows.

### 3.2. Wireless Communication Technologies

Plenty of wireless technologies are available to deploy smart healthcare systems, each one with its own properties in terms of radio coverage, data transmission rates, frequency, latency, power consumption, etc. All these features must be considered when envisaging smart health solutions. The landscape of wireless communication technologies for smart healthcare is described in what follows, and a comparison between these technologies and their suitability in the aforementioned WBAN architecture is summarised in Table 11 and Table 12.

#### 3.2.1. Bluetooth

One of the most popular short-range wireless communication technologies is Bluetooth (see Table 11). Previously standardised under the IEEE 802.15.1, currently known as the Bluetooth Special Interest Group, it has established its specifications and developments. Bluetooth enables transmitting data between two wireless devices, one of them acting as a master (commonly the sink) and the other as a slave, in a range of, at most, 100 m at a data rate up of to 3 Mbps. This technology operates in the 2.4 GHz ISM band, also used by Wi-Fi and ZigBee technologies, and frequency hopping-related techniques were applied to reduce potential interference. This technology reached its popularity in the early 2000s with the emergence of mobile devices, and even today is extensively used in numerous general-purpose portable devices, including smartphones, smartwatches, fitness trackers, laptops and computer peripherals. However, with the advent of resource-constrained devices, a very low-power Bluetooth specification was developed: BLE (see Table 11) [15,18,249], able to transmit data with a very low power consumption and latency at 2 Mbps in a range of 400 m. The BLE characteristics are well aligned with s-health applications, such as critical emergency response, so to communicate with wearables, IoT, IoMT and other devices deployed in WSNs and WBANs. For instance, this technology has been a great ally to develop contact tracing applications during the COVID-19 outbreak [250]. Hence, we foresee BLE as an excellent technology for the next-generation medical purpose oriented devices.

#### 3.2.2. ZigBee

ZigBee [8,18,249], designed by the ZigBee Alliance and built on the IEEE 802.15.4 standard, is another outstanding wireless technology (see Table 11). This technology was advantageous for its low power consumption, so battery-powered devices can be operational for several years before battery depletion. Different characteristics in terms of coverage, data rate, power consumption and operational frequency bands are offered upon the selected ZigBee module, with XBee being the simplest one. The coverage radio is generally up to 100 m (similar to Bluetooth’s), but data are transmitted at a low data rate up to 250 kbps. Hence, ZigBee might not be suitable for transmitting user-centric data in real-time which requires immediate action. Notwithstanding, this technology could be considered for battery-powered IoT devices oriented towards contextual sensing in WSNs.

#### 3.2.3. IEEE 802.15.6

IEEE 802.15.6, the latest international standard for WBAN communications, is oriented towards short-distance communications between devices operating on, in or around the human body [8,18,251]. The standard defines three physical layers, each operating at different frequency bands for different purposes (see Table 11). First, the narrowband (NB) comprises seven frequency bands between 400 MHz and 2.4 GHz with low data rates of up to 900 kbps (e.g., the 400 MHz band is used for implant communication and the 600 MHz band for medical telemetry). Second, the ultra-wideband (UWB) operating at higher frequencies between the 3.2–4.7 GHz and the 6.2–10.3 GHz band enable higher data rates of several Mbps (up to 15 Mbps) between on-body devices and on/off-body devices, such as for entertainment systems. Third, the human body communication (HBC) using the human body as a channel operates in low bands between the 14–18 MHz and the 25–29 MHz and transmits data at a maximum rate of 2 Mbps in an energy-efficient way.

In addition to the low power requirements, communications must be reliable, considering that devices are continuously changing their location due to humans’ movements. Furthermore, regarding securing communications, three security levels are defined [252]: level 0 does not provide any security mechanisms and unsecured communications are established, level 1 provides message authentication and integrity assurance, but no encryption mechanisms, and level 2 provides message authentication, integrity assurance and encryption. This standard is expected to be adopted by miniaturised and resource-constrained medical devices to properly communicate the user-centric data.

#### 3.2.4. Wi-Fi

One of the most used general-purpose wireless technology is Wi-Fi, available in most devices from the digital ecosystem [18,249]. Wi-Fi encompassed within the IEEE 802.11 standards family for wireless communications in local area networks, is suitable for transmitting large volumes of data in a range of tens of meters at very high data rates (in the order of Mbps or even Gbps at latest specifications), where power consumption is not a critical issue (see Table 11). IEEE 802.11n, also known as Wi-Fi 4, operates in the frequency band between 2.4 GHz and 5 GHz and supports a theoretical data rate of up to 600 Mbps. IEEE 802.11ac (Wi-Fi 5) exhibits better performance and better radio coverage compared to its predecessors, operating in the 5 GHz band and providing data rates from 400 Mbps up to 1 Gbps. Recently introduced, the latest specification IEEE 802.11ax (Wi-Fi 6) increases data rates up to 10 Gbps, strengthens security with WPA3 and reduces the energy consumption compared to its predecessors, hence opening the door to its possible use in some resource-constrained devices in the coming years [253]. These characteristics make Wi-Fi technology a suitable solution for large-scale real-time smart health services.

#### 3.2.5. Cellular Networks

The tremendous popularity of smartphones during the last decade motivated the evolution of cellular networks, originally devoted to providing telephony services, towards high-bit rate transmissions of data. Today, the LTE-based 4G technology is available in many off-the-shelf smartphones and other portable devices (see Table 12). This technology operates at different bands between the 700 MHz and the 2.6 GHz frequencies (different among countries) and supports high data rates of hundreds of Mbps at a relatively low latency. Similarly to Wi-Fi, the main limitation of 4G is its high energy consumption, which limits its implementation in resource-constrained devices, although most current smartphones and smartwatches (i.e., the sinks) implemented this technology. Fourth Generation (4G) technology perfectly fits long-range communications in the outdoors, where secure Wi-Fi access points are less available.

The fifth generation of mobile networks, 5G, has undoubtedly been one of the main buzzwords of recent years. Expected to enable the massive deployment of IoT in a truly connected world with billions of devices [254], 5G promises very high data rates of up to several Gbps (especially at higher frequency bands of millimetre waves) in an almost negligible latency (1 ms ideally), using only a fraction of the energy consumption of 4G (see Table 12). To make 5G a reality, lots of antennas will need to be installed in order to manage an unprecedented coverage density of approximately a million devices per square kilometre. However, this requires a substantial investment in infrastructure. 5G will certainly open the door to numerous s-health opportunities, even though some of them could sound futuristic today, such as augmented/virtual reality assistance for blind people, remote collaboration in surgical interventions or video-enabled medication adherence [255,256,257].

#### 3.2.6. Low-Power Wide-Area Networks

Long-range communications can be hardly implemented in sensors and IoT devices due to its aggressive power consumption. To fill this gap, the low-power wide-area networks (LPWANs) emerged as a novel communication paradigm (see Table 12). These kinds of technologies are able to transmit data along large distances (up to several kilometres) at a very low power consumption. However, these communications were conducted at a low data rate and high latency [258,259]. These technologies are hence not suitable for real-time applications, although they could be adopted for contextual sensing, whose values vary slightly over time and real-time constraints are relaxed or for non-critical healthcare monitoring, such as rehabilitation.

Some of the most prominent LPWAN technologies are LoRa, SigFox andNB-IoT [15,249,255,260]. In short, SigFox is an easy-to-deploy technology enabling large network connectivity at low infrastructure costs. However, the data transmission rate is very low (between 100 and 600 bps) and the latency is the highest in comparison to similar technologies. Interestingly enough, LoRa offers an excellent trade-off between distance coverage, data rate and energy consumption, and its popularity in IoT arenas has significantly grown in later years and it is expected to grow further. NB-IoT, although enhancing LoRa’s properties in terms of latency and data rate, is scarcely adopted in IoT devices and lacks deployment readiness.

#### 3.2.7. Other Technologies

In addition to the aforementioned wireless communication technologies, there are further technologies that could be well suited for smart health purposes. RFID and NFC are popular solutions for very short-range communications, particularly interesting for indoors. Other promising low-power technologies that could complement or even replace ZigBee or Bluetooth in the coming years are, among others, Z-Wave, ANT and RuBee. Within the LPWAN-related standards, weightless could be an interesting solution for communicating devices in the industrial and medical field. Finally, WiMAX (IEEE 802.16) could contribute to establishing the long-range transmissions of several kilometres where energy consumption is not critical.

### 3.3. Evaluation of Wireless Technologies

Many wireless technologies are available for deploying smart healthcare solutions. As shown in Table 11 and Table 12, each of them is suitable at different tiers of the aforementioned WBAN architecture due to its inner characteristics of radio coverage or power consumption, among others. However, to select the most adequate technology for each tier, other aspects must be considered.

One of the most important characteristics is the throughput of each technology, whose evaluation is not straightforward. Although the throughput could naively be approximated to the data rate, it diminishes in real environments due to interference or packet losses. For instance, whereas SigFox and LoRa enable high interference resilience, NB-IoT lacks interference immunity [261]. Therefore, although NB-IoT has a higher data rate than the other LPWAN technologies, a non-negligible number of frames could be lost and decrease its throughput consequently. Another key aspect to consider is the message size and the message frequency, i.e., the payload capacity. For instance, SigFox messages can carry a payload of 12 bytes and SigFox limits each SigFox device to 140 messages per day [262]. Finally, media access control also plays a crucial role in a network’s throughput. For example, the RTS/CTS mechanisms can be used by 802.11 standards’ family to avoid transmission collision, but at the price of lowering its throughput, especially in dense networks. Finally, scalability is another fundamental aspect once deploying smart healthcare systems intended for large populations. Whereas ZigBee and LPWAN technologies have a great scalability [261], scalability in 4G and 5G networks depends on the density of base stations deployed, and on the number of sinks and access points in BLE and Wi-Fi networks.

## 4. Information Security: Requirements, Attacks and Solutions

Security and privacy issues are critical concerns in any type of information system. However, these issues are even further strengthened in smart healthcare systems due to the high confidentiality of the information managed. More specifically, security and privacy aspects must be considered throughout the entire system: from the very sensing devices where data are collected, through the network where the data are transmitted, to the HIS where data are stored, analysed and presented to end users.

This section addresses the issue of information security in smart healthcare services from a global scope, by describing the security requirements that all s-health systems must fulfil in Section 4.1, categorising the most common attacks in these systems in Section 4.2, and proposing appropriate solutions to avert those attacks in Section 4.3.

### 4.1. Security Requirements

As any other information system, smart healthcare systems must pursue a number of security requirements and put in place the appropriate protection mechanisms to guarantee them. Then, the main security requirements that must be considered are briefly discussed:

- Confidentiality: Data confidentiality is the property that guarantees that data are only disclosed to authorised entities (e.g., people, devices, processes…), whilst remaining unintelligible to unauthorised entities. User-centric data, but especially the medical, must be kept confidential during storage periods (susceptible to data leakages) and while being conveyed through the communication networks (susceptible to eavesdropping). The most widely used technique to achieve confidentiality is encryption, in which only authorised entities have access to the secret key required to decode the data.
- Integrity: Data integrity ensures the accuracy, trustworthiness and completeness of data, guaranteeing that the data have not been modified or destroyed by unauthorised entities. For instance, attackers might tamper the data without authorisation during its transmission over the network. Unless properly detected, smart healthcare systems would react to users upon faux data, and potentially endanger their health. Moreover, other non-related human events can also threat integrity, such as hardware glitches. Integrity-oriented protections include cryptographic hashes for detecting data modifications, and redundancy and backup policies enable restoring any affected data if necessary.
- Availability: Data availability guarantees that authorised entities have constant access to the data regardless of their location and time. This property allows the proper functioning of the sensing devices, the communication channels and the information systems at a whole. Smart healthcare systems must guarantee the availability of medical data, since decisions might be made anytime and anywhere. Hence, they must be resilient to service disruptions: either intentional from attackers denying services to legitimate users, or accidental due to natural disasters, hardware failures or system upgrades that require systems breakdowns. Redundancy, recovery policies and fail-over strategies should be considered to avoid availability issues.
- Non-repudiation: Non-repudiation is the guarantee that a particular interaction between two entities actually occurred. This means that, given the communication of a message between two authorised entities in a system, the sender cannot deny having sent a message to the receiver in the future, and the receiver cannot deny having received the message from the sender in the future. Although cryptographic digital signatures can help achieve this property, it is noteworthy that their use in some sensing devices might be limited due to their computational constraints.
- Authentication and authorisation: Authentication and authorisation mechanisms are commonly misconceived or interchanged. On the one hand, authentication refers to the process of confirming the identity of an entity, i.e., determining whether the entity is who it claims to be. On the other hand, authorisation refers to the process of determining whether the authenticated entity has access to the particular resources and services of the system. Within smart healthcare systems, authentication procedures are mandatory in order to establish communications only with properly authenticated entities, and avoiding any communication with illegitimate entities. In general, this is achieved through credentials, e.g., passwords, biometrics or digital certificates. In the case of successful authentication, then systems must ensure whether the entities have permission to do the actions that aim to (e.g., access, modify or delete medical information).
- Privacy: Privacy is a fundamental right that has to be protected. Smart healthcare systems must process personal data in a lawful, fair and transparent manner for a specific, limited and legitimate purpose. Besides, due to the sensitivity of the data, they require the explicit individuals’ consent for their managing and be compliant with the current regulations on data privacy. These systems must adopt the appropriate safeguards to reduce disclosure risks, including identity disclosure, i.e., the direct re-identification of individuals, and attribute disclosure, i.e., the inference of confidential information to a certain individual. Hence, in the case of data leakages or eavesdropping, people’s privacy is not jeopardised. One of the most common data sanitisation techniques for privacy protection is data anonymisation.

### 4.2. Security Attacks, Threats and Vulnerabilities

The impact of attacks against smart healthcare systems may go beyond the leakage of medical records and the loss of privacy, and life-threatening situations may arise in the case of hijacking implantable devices, such as insulin pumps or pacemakers [263,264,265,266]. Unfortunately, attacks against vulnerabilities discovered in medical devices are unexceptional [267,268]. In addition, wireless technologies and the proper HIS may also entail a number of security flaws depending on the design and implementation of the system: insecure programming practices, vulnerable communication protocols or obsolete technologies open the door to numerous security attacks [269,270,271].

As observed, smart healthcare systems are susceptible to different types of security attacks. In the literature, different taxonomies were proposed to classify them. Then, the most popular classification methods are outlined:

- Based on the attack’s nature: passive attacks and active attacks [21]. In passive attacks, attackers monitor and collect information from the system and exploit it to launch further attacks. This kind of attacks does not harm the system, hence victims are not aware of them. On the contrary, active attacks are intended to modify or damage the system by injecting, altering or destroying data or services. Since these attacks impact the systems, victims are informed of them.
- Based on the attack’s origin: internal attacks and external attacks [21]. Internal attacks are initiated by malicious entities located inside the system, i.e., insider attackers. In contrast, external attacks are launched by external entities located outside the system, i.e., outsider attackers.
- Based on the attack’s launch method: physical methods, logical/software-based methods and side-channel methods [272]. Physical methods refer to the attacker’s ability to have physical access to the cyber-physical system in an unauthorised way. Logical or software-based methods exploit vulnerabilities and expose errors in logical systems, such as software, operating systems, applications or protocols, to gain illegitimate access. Side-channel methods observe the indirect physical effects of the systems during their functioning to acquire advanced knowledge.
- Based on the TCP/IP model layer: application layer, transport layer, network layer and network interface layer [272]. Attackers can target different layers of the TCP/IP model to find weaknesses and infiltrate the system. Similar classifications can be performed using the OSI model.

**Figure 5:**
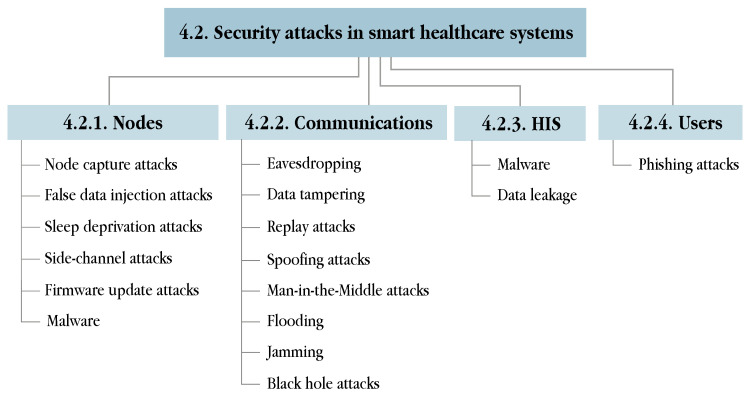
The taxonomy of security attacks in smart healthcare systems.

Complementing the taxonomies above, this article presents a classification of security attacks based on the actors involved in the smart healthcare systems, namely nodes, communications, HIS and users. It is noteworthy that the list of attacks below is not exhaustive, and only covers the most widely known attacks related to smart healthcare. In short, Table 13 classifies the security attacks reviewed in this article according to the aforementioned taxonomies, and indicates which security requirements are compromised as a consequence. In addition, a graphical summary is provided in Figure 5.

#### 4.2.1. Attacks against Nodes

The resource-constrained nature of most of the nodes deployed in WBANs and WSNs limits the incorporation of robust security mechanisms. Hence, nodes become the primary target of many attackers. In particular, attacks related to node compromising, including node capture attacks, false data injection attacks and sleep deprivation attacks are explained. Furthermore, side-channel attacks and firmware update attacks are also considered within this scope.

##### Node Capture Attacks

One of the most popular attacks is node capture attacks, in which attackers take control of a node after successfully exploiting a vulnerability [273,274,275]. These attacks need to be rapidly detected to disconnect the compromised node from the network as soon as possible. Otherwise, attackers may seek for further vulnerabilities within the system to elevate their privileges and eventually, gain control over the entire system. These attacks mainly compromise the confidentiality of the system because attackers could extract the private information from the captured node, such as user-centric data in the case of wearable devices or the cryptographic keys stored in the nodes to encrypt and decrypt the communications. Within the s-health scenario, this attack can indirectly threaten people’s privacy because raw sensitive data might be disclosed to attackers.

##### False Data Injection Attacks

Once a node has been compromised, attackers can inject malicious code in the captured node and redeploy it in the network (as if it was a legitimate node) with the aim to perform unintended functions. Usually, attackers can use the captured node to conduct false data injection attacks, i.e., fabricating erroneous data as if they were true or preventing passing true data [276]. Hence, the integrity of smart healthcare systems could be compromised because they would naively react to fake data and take unsuitable health decisions that might put people’s lives at risk [277,278]. For example, the injection of false physiological parameters could lead to wrong medical diagnosis and consequentially to inadequate medical treatments based on these data. The severity of these attacks increases in critical operations such as surgeries, where the injection of real-time parameters could result in a loss of life. Furthermore, false medical records may cause illegal insurance claims, thus opening the door to potential financial fraud.

##### Sleep Deprivation Attacks

More aggressive attacks which damage (either physically or logically) the sensor network and disrupt network communications are sleep deprivation attacks (also known as energy drain attacks) [279,280]. These attacks aim to increase the power consumption of captured nodes with useless tasks, such as running infinite loops, so as to accelerate the battery draining of the devices and hence, force their disconnection from the sensor network. By disconnecting nodes, the system’s availability becomes affected, and dramatic consequences could arise within the healthcare domain. In particular, stopping vulnerable life-assistance devices, such as pacemakers [281] or cardiac defibrillators [282], can impact human lives.

##### Side-Channel Attacks

Whereas most attacks aim to exploit the weakness of algorithms and protocols implemented in the nodes, the family of side-channel attacks concentrate on exploiting the physical effects of computing devices during their normal functioning to infer sensitive information, namely cryptographic keys and passwords [283]. Such attacks can leak relevant information about these devices through physical side signals, such as timing analysis (i.e., time taken to perform computations), power analysis (i.e., variations on the power consumption to perform computations), electromagnetic emanation (i.e., radiation emitted by the system to perform computations) and acoustic attack (i.e., sounds produced during computations), among others [272]. All in all, side-channel attacks are difficult to handle and pose serious threats due to their non-invasive nature, the generally passive mode, and the fact that they evaluate the physics, rather than the implementation, of the computing elements.

The numerous nodes deployed in smart health systems open the door to a plethora of side-channel attacks [284]. For instance, several studies have shown the feasibility of inferring the key-based security system of smartphones or smartwatches by means of motion sensors [285,286,287]. In this scenario, attackers would gain the additional advantage of bypassing the security mechanisms defending the system and thus, break into it more easily. More active side-channel attacks might also put people’s lives at risk, such as by injecting electromagnetic signals that might bogus the legitimate signals of cardiac implantable devices [288].

##### Firmware Update Attacks

Modern nodes require firmware updates to support the latest technological developments and improve performance. For time inefficiencies, these updates are no longer performed physically between the manufacturer and the device, but remotely, in which the devices are able to automatically download the latest firmware version and upgrade themselves. However, the security of firmware updates is generally insufficient due to the lack of encryption and/or authentication mechanisms [289], opening the door to firmware update attacks. This attack consists of injecting a malicious firmware into the vulnerable device so as to grant attackers total control over them. The main severity of these attacks is that they are capable of affecting entire families of nodes, e.g., if a manufacturer uses the same firmware update mechanism, then all its devices would be vulnerable. Particularly, numerous commercial wearable devices, especially fitness trackers, are susceptible to this kind of attacks due to their computational constraints [290,291,292,293,294]. Even worse, vulnerabilities in the firmware update procedure, able to execute arbitrary code, of automated external defibrillators and implantable devices have been reported [265,295].

#### 4.2.2. Attacks against Communications

The distributed nature of smart healthcare systems requires communication networks to transmit the information between the different actors within the system. Most of the existing attacks aim to target the communications, mostly wireless, which are prone to hijacking unless properly secured. Eavesdropping, data tampering, replay attacks, spoofing attacks, man-in-the-middle attacks and denial of service attacks are subsequently described within the s-health paradigm.

##### Eavesdropping

Continuous communications among entities are exposed to be intercepted by eavesdrop attackers. Eavesdropping (or sniffing) attacks aim to secretly capture and listen to the data packets transiting the communications, without the knowledge of the legitimate entities [296]. During eavesdropping, all messages are compromised and attackers can analyse the traffic to learn private information from the whole system (e.g., data, protocols, communicating entities…) [297]. For this reason, messages must never be transmitted in plain-text or encrypted with vulnerable algorithms. This passive attack undermines the confidentiality of the communications and might jeopardise people’s privacy.

Some Bluetooth and BLE communications, extensively used in the s-health domain, might be vulnerable to eavesdropping attacks, where encryption might be bypassed [298,299,300]. Indeed, a variety of medical devices, including hospital equipment [301], wearables [302,303,304,305,306] and implantable devices [282,307], have been compromised through eavesdropping, by disclosing private data or serving to obtain insights for further active attacks. Eavesdropping attacks are also common in other popular technologies, namely Wi-Fi and ZigBee [308,309].

##### Data Tampering

More actively, attackers can deliberately alter or destroy data transiting through the network. This attack, commonly known as data tampering or modification attack, aims to compromise the integrity of the data and as a consequence, the system’s [310]. By means of data tampering attacks, attackers could modify the data at their convenience in order to manipulate the system’s functioning or gain access to it. For instance, systems would malfunction in the case of modifying data packets properties, such as their timestamps (i.e., the flow of events would be erroneous from the system’s perspective) or their destination address (i.e., redirecting them to illegitimate destinations) [311].

More threatening, the unauthorised modification of more sensitive data, such as medical, could cause physical damage on people’s health, because systems would react upon malicious data. This situation might lead to overtreatment, undertreatment or even death [312]. Several data tampering attacks have been successfully conducted using medical equipment [301,311], fitness trackers [291,293,306,313] and even implantable devices [265,266,314]. No less important, tampering with contextual data can also negatively impact the lifestyles of large populations, as smart healthcare systems would adapt themselves to false contextual conditions covering a certain geographical area. In short, tampering user-centric data leads to individual damages, but tampering contextual data might lead to large-scale damages.

##### Replay Attacks

During eavesdropping, attackers capture valid data packets that are sent between two legitimate entities. Even though their messages could be encrypted (and thus unreadable for attackers), these messages have an effect on the recipient entity. Attackers can exploit this to mislead legitimate entities and acquire the trust of the system with the aim to maliciously duplicate transactions, impersonate entities or raise confusion within the system. Thus, replay attacks occur when unauthorised entities re-send legitimate captured data packets at a later time while acting as the original sender, hoping to repeat some action that benefits the attacker [315]. For instance, if attackers intercept the messages related to a valid login procedure, they could try to replay them later on and, unless detected by the system, obtain access to the system without knowing the actual credentials. This attack clearly threatens data freshness, another important attribute in information systems [316].

The consequences of successful replay attacks in smart healthcare systems can be tragic, especially when replaying messages describing users medical data [317,318,319,320]. Systems would naively react to old physiological parameters rather than to the current physiological parameters of users in that moment. Replaying old messages for a long period of time may bring mistreatment. Among others, studies have demonstrated the possibility of targeting diabetic people by launching replay attacks with false glucose readings [321] or exploiting the validation limitations regarding integrity and authentication in cardioverter-defibrillator devices [282] and other implantable medical devices [263,266]. Similarly, replay attacks were also detected in a number of fitness trackers due to the lack of authentication mechanisms [293].

##### Spoofing Attacks

Identity theft is undoubtedly one of the primary security concerns in information systems. Spoofing attacks consist of masquerading attackers acting as legitimate entities by using forged data [322]. If the legitimate entities within the system trust in the incoming (malign) entity, attackers can gain access to once inaccessible resources, and conduct further insider attacks. There exist different spoofing attacks targeting different OSI layers, namely ARP spoofing (i.e., attackers link their MAC address to a legitimate network IP address), IP spoofing (i.e., attackers send IP packets from a spoofed source IP address to disguise themselves) and DNS spoofing (i.e., attackers re-route specific domain name requests to different IP addresses under their control) [323,324].

The computational, size and power limitations of most nodes prevent the implementation of spoofing countermeasures. Hence, these devices may be vulnerable to spoofing attacks, hence compromising the entire s-health system. Successful spoofing attacks enable attackers retrieve medical data gathered from the sensing devices, such as fitness trackers [294] and even trigger life-threatening situations in the case of spoofing insulin pumps [325]. Beyond medical data, the ability to spoof GPS data was also pointed out [326,327], forging the real location of devices (and hence, the users as well).

##### Man-in-the-Middle Attacks

One of the most popular and devastating attacks in networks are man-in-the-middle attacks (MitM) [328]. Such attacks consist of intercepting the communication between two legitimate entities who believe that they are directly communicating with each other. Once a communication is hijacked, attackers are free to passively eavesdrop the data packets seeking for private data or actively manipulate the data packets by tampering data or injecting false data. Moreover, attackers can exploit this illegitimate advantage for redirecting traffic to malicious resources or spreading malware through the network. With the lack of security of many sensing devices, MitM attacks can be feasibly exploited in sensor networks [329,330]. Indeed, some Bluetooth communications have been proven to be vulnerable to MitM attacks [299,331,332]. As a result, there is a need to evaluate the trustworthiness of entities within sensor networks so as to ensure the confidentiality and integrity of the transmitted data through such networks [333,334,335].

The impact of MitM attacks on smart healthcare systems can be tremendous. Attackers could intercept medical records shared between two legitimate healthcare providers, or intercept physiological data collected from sensing devices without the knowledge of the actors. Numerous nodes are vulnerable to this kind of attack due to the security flaws and weak authentication mechanisms. For instance, the lack of encryption of some devices enables attackers to seamlessly hijack communications and capture private data such as session identifiers, passwords and health data [291,293,303,305,313,336,337,338]. Of further concern, studies HAVE also discovered MitM-enabling vulnerabilities in protocols integrated in implantable medical devices [339,340].

##### Denial of Service Attacks

Attacks against availability prevent the normal performance of information systems and threaten network functioning and resources responsiveness. Denial-of-service attacks (DoS) aim to make resources unavailable to the legitimate users by temporarily or indefinitely disrupting the services provided [341]. A more sophisticated version is that of distributed denial-of-service attacks (DDoS) which require multiple and coordinated sources controlled by an attacker targeting a victim, who cannot stop the attack by just blocking a single source. In addition to the aforementioned sleep deprivation attacks at the node level, there exist further DDoS-oriented attacks at the communication level. Usually, the most prominent attack is flooding which overwhelms legitimate resources with purposeless requests, in such a way that they are not able to handle all the incoming packets (even the legitimate ones) and then collapse. Attackers can flood the network with data packets from different layers of the OSI model, such as HTTP flood, ICMP flood, SYN flood, DNS flood and HELLO flood. Another well-known type of DDoS attack is jamming, which uses specific jammer devices to generate random radio-frequency signals that deliberately cause interference and hence, disrupt the network’s functioning [342]. Another DDoS attack at the network layer is the black hole attack (also called packet drop attack), where a malicious node exploits vulnerabilities in routing protocols to redirect the traffic towards itself and then, drop the incoming packets [343].

Many DDoS attacks have been launched in the recent years with the Mirai botnet, which exploits the low-security implementations of IoT devices to disrupt services [344,345]. This kind of disruptive attack could lead to terrifying consequences in smart healthcare systems, which need to be constantly on for real-time monitoring and act immediately in the case of emergencies [293,303]. Unfortunately, availability is particularly crucial in certain critical-mission medical devices, such as implantable devices, wherein availability-threatening attacks can lead to the loss of people’s lives [266,282,346].

#### 4.2.3. Attacks against HIS

Certain security attacks aim to target the very HIS infrastructure of the healthcare service providers, i.e., the servers, databases, routers, firewalls and computers that manage the system’s applications, data and flows. These attacks are likely to be more sophisticated since these systems, which are not resource-constrained, are able to implement more robust countermeasures. More specifically, malware and data leakage attacks are outlined as follows.

##### Malware

Malware, short for malicious software, encompasses all different types of unwanted and hostile programs, used to invade, damage, disrupt or disable computer systems and networks [347]. This creates chaos and compromises confidentiality, integrity and availability, by exploiting the vulnerabilities of the systems [348]. Infected systems are partly (or completely) under the control of attackers and therefore are susceptible to data theft, hijacking and propagating the malware into other systems. Malware can be divided into different categories, including worms, trojans, rootkits, viruses, spyware, keyloggers, botnets and ransomware [329]. In particular, ransomware attacks have become popular in recent years with the aim of hijacking a system whose files are encrypted with an attacker’s key, and ask for some payment in cryptocurrencies to restore them [349].

For decades, many malware have been developed to target conventional computing devices. However, with the advent of sensing devices, wearables and IoT devices, different malware variants emerged to target these more vulnerable devices. In particular, the lack of strong security mechanisms makes these devices highly vulnerable to malware infection [293,301]. The Mirai botnet, VPNFilter, BrickerBot or Reaper are some examples of malware targeting large networks of IoT devices [329,344,345,350]. In addition, the popular WannaCry ransomware-based attack threatened millions of organisations in 2017 and hijacked multiple healthcare systems, including the British National Health Service [351]. Hence, smart healthcare systems must monitor the functioning of their entities, seeking for abnormal malware-derived conditions, and, once detected apply the proper countermeasures to prevent the malware spread and mitigate the impact on the entire system.

##### Data Leakage

Data leakages (or data breaches) occur when personal and/or confidential data from an organisation is released to an untrusted environment by an unauthorised entity. Most data leakages involve financial information, medical records, trade secrets or intellectual property, whose value on the black market may be significant [13]. Unfortunately, these incidents are far too common [352,353,354]. These attacks constitute a threat to the confidentiality and privacy, essential principles that users expect from organisations once managing their personal information. It is noteworthy that leakages are not only due to malicious attacks, but they may also be due to unintentional human actions (e.g., unintentional emailing to wrong recipients) or system glitches. However, this does not extinguish organisations from legal liabilities and economical sanctions, in addition to the considerable reputational damage.

#### 4.2.4. Attacks against Users

The weakest link in the security chain of a system is the human factor [355]. Hence, attackers can attempt to infiltrate themselves into target systems by taking advantage of the lack of knowledge of most users regarding computer security, instead of seeking for vulnerabilities in their infrastructure, network and nodes. To this aim, attackers conduct social engineering activities to exploit humans error, among which phishing attacks are the most prominent.

##### Phishing Attacks

Phishing attacks aim to deceive users and obtain sensitive data wherein attackers disguise themselves as a legitimate entity [356]. The most common phishing is by e-mail, where attackers forge the sender’s address to seem legitimate, hoping that users trust it and introduce private data or download some malware. In the case of successfully deceiving users, then attackers can impersonate the victims, acquire confidential data from the systems or launch further malicious activities on their behalf for raising their privileges. Phishing has evolved towards more sophisticated attacks, namely spear phishing (i.e., targeted phishing attacks using OSINT tools), whaling (i.e., targeted phishing attacks to high-privilege corporation’s users), vishing (i.e., voice phishing) or smishing (i.e., SMS phishing), among others. With the worldwide COVID-19 pandemic, attackers have used the opportunity to intensify phishing campaigns for deceiving users with fraudulent messages [357,358,359]. Phishing attacks in the healthcare domain are common: attackers can impersonate legitimate users and gain access into the HIS [360,361,362]. In such cases, systems should be able to detect abnormal accesses (e.g., the source IP address or country is not the usual one) and prevent the entrance of users into the system unless performing a second authentication.

### 4.3. Security Solutions

**Figure 6:**
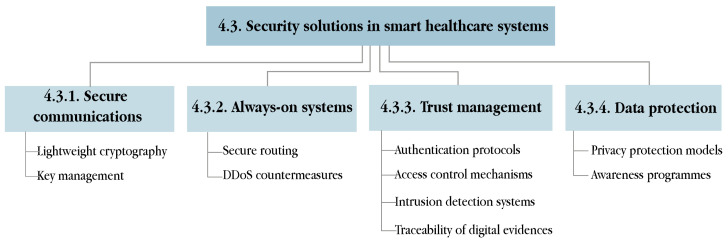
Taxonomy of security solutions in smart healthcare systems.

Achieving a high level of security in smart healthcare systems is challenging because this requires the implementation of many security mechanisms at different layers. Then, the most popular security solutions and safeguards to be considered in these systems are described. For the sake of clarity, these solutions were classified into four groups: secure communications, always-on systems, trust management and data protection (see Figure 6). Moreover, Table 14 summarises the proposed solutions and indicates which security requirements are protected when adopting them.

#### 4.3.1. Secure Communications

Many security attacks are communication and network oriented. Although securing these channels is always paramount, it is of utmost importance in smart healthcare systems. To this end, all the entities involved in the storage and transmission of data must consider the use of cryptography. Solutions based on lightweight cryptography and key management are discussed below.

##### Lightweight Cryptography

Servers, desktop computers, tablets or smartphones are powerful enough to implement state-of-the-art cryptographic solutions. However, conventional cryptography is unsuitable for resource-constrained devices. Hence, they must rely on lightweight cryptography [363,364], which focuses on the design of simpler and faster cryptographic primitives, standardised under the ISO/IEC 29192 [365].

In recent years, many symmetric key solutions were proposed to ensure confidentiality [366]. Whereas AES remains the standard algorithm in conventional cryptography, many block ciphers have simplified their properties for lightweight cryptography (i.e., smaller block sizes, key sizes and number of rounds) in order to improve their efficiency. PRESENT, CLEFIA and LEA are the current block cipher algorithms within ISO/IEC 29192-2:2019 [367,368,369]. Although lightweight stream ciphers can be used in constrained environments, they are less prominent compared to block ciphers. The two standardised algorithms in ISO/IEC 29192-3:2012 are Enocoro and Trivium [370,371].

Regarding public-key cryptography, lightweight techniques based on Elliptic-curve cryptography (ECC) have been presented [372]. In general, ECC-based implementations are more efficient compared to classical approaches such as RSA, since ECC can reach the same level of security with significantly shorter key lengths and moreover, are not based on computationally demanding complex operations [373,374,375,376]. For instance, TinyECC is a configurable and publicly-available ECC library suitable for supporting public-key cryptography in sensor networks and IoT devices [377]. With a view on the post-quantum era, where RSA and ECC algorithms can be vulnerable, lattice-based cryptography [378] is becoming increasingly important and its feasibility in lightweight IoT devices is promising [379,380].

Cryptographic primitives can also provide data integrity assurance by means of hash functions, which help determine integrity-oriented attacks. However, conventional hash functions, such as SHA-2 and SHA-3, might not be efficient enough for constrained devices, and lightweight hash functions using shorter messages and outputs have been proposed. PHOTON, SPONGENT and Lesamanta-LW are standardised within ISO/IEC 29192-5:2016 [381,382,383]. Furthermore, lightweight message authentication codes (MACs), used to verify the authenticity and the integrity of the message have also been defined in ISO/IEC 29192-6:2019: LightMAC and Chaskey [384,385].

##### Key Management

As long as the cryptographic keys are securely managed, the security of the communications is guaranteed. Key management deals with the generation, exchange, storage, use and revocation of the cryptographic keys in a distributed system. In particular, smart healthcare systems must deploy robust key management policies to safeguard confidentiality.

Randomness is an important factor in computer security, since the generation of cryptographic keys requires random values to ensure their uniqueness and unpredictability. The implementation of random number generators in resource-constrained environments is challenging due to the hardware and software limitations, despite the fact that the design of lightweight algorithms is currently on the rise [386,387,388]. In the specific context of wearable devices, different private key generation schemes have been proposed based on the randomness of user-centric attributes, including heart rate [389,390,391,392,393] and body motion [394,395].

#### 4.3.2. Always-On Systems

Smart healthcare systems must be uninterruptedly available. However, due to the increasing sophistication of attacks against systems’ availability, fulfilling this requirement is challenging. More specifically, this section addresses two major solutions against systems’ availability, namely secure routing and DDoS countermeasures.

##### Secure Routing

Routing is fundamental to enable communications in any kind of network. In smart healthcare, routing information must be properly communicated in a time and energy-efficient way [247,396]. As nodes can join and leave the network on-the-fly (e.g., a new sensor is introduced, a sensor has crashed, or a sensor has been compromised and removed from the network), routing solutions must be autonomous, scalable and dynamically adapt to these changes [397,398]. Routing protocols must also be resilient to attackers who can inject malicious routing information into the network (e.g., when a node is captured) to cause routing inconsistencies and disrupt communications. Numerous studies have been defined to secure routing protocols within sensor networks, based on the trustworthiness of neighbour nodes, clustering or hierarchical methods and genetic evolutionary techniques [399,400,401,402].

##### DDoS Countermeasures

Different defensive DDoS countermeasures must be adopted at different stages to avert these attacks, categorised into preventive measures, detection measures and responsive measures [341,403].

Prevention mechanisms are intended to decrease the probability of suffering DDoS attacks. These methods can be generally classified as filtering-based or capacity-based [404]. Filtering-based mechanisms aim to decrease the network traffic by distinguishing legitimate traffic from attacking traffic, which is dropped. To do this, the IP traceback method determines the true IP origin address of a data packet, rather than its spoofed IP address. Probabilistic/deterministic packet marking, route-based packet filtering, history-based IP filtering or ingress/egress filtering approaches are used to filter data packets using different criteria, such as their source or destination address or their reachability [405,406,407].

The monitoring of the system’s metrics serves to detect abnormal behaviour. Under DDoS attacks, these metrics are abnormal because the system would be overwhelmed and would degrade its quality of services. For years, most DDoS detection methods were based on statistical analysis, but several machine learning solutions have recently been proposed. Statistical methods can effectively and efficiently detect DDoS attacks by monitoring the incoming traffic at different time periods through entropy, principal component analysis and hidden Markov models [408,409]. With regards to machine learning techniques, many classifiers have succeeded in identifying DDoS attacks (e.g., support vector machines, neural networks, random forest…) from data packets’ features, such as their size, origin and destination addresses, ports, protocols or time interval between them [410,411,412].

Finally, but no less importantly, the detection of DDoS attacks must be immediately followed by a proactive response. Smart healthcare systems must be designed to be fault-tolerant and limit DDoS damage. Among other strategies, scaling hardware resources, queuing techniques or migration-enabling services should be considered.

#### 4.3.3. Trust Management

Systems require mechanisms to be trustworthy, i.e., by producing reliable and authentic data and communications and being accountable. Implementing authentication protocols and access control mechanisms, considering intrusion detection systems and tracing digital evidence are some solutions to this aim.

##### Authentication Protocols

Authentication is paramount to prevent disclosing information to unauthorised entities. Indeed, both users and devices must be authenticated in smart healthcare systems. Traditionally, user authentication mechanisms were password based. However, weak passwords or the systematic reuse of passwords are common malpractices that facilitate the task of attackers to overcome these authentication mechanisms. Current authentication mechanisms combine possession factors (e.g., smart cards, one-time password tokens), knowledge factors (e.g., passwords, PINs) and biometric factors (e.g., fingerprint, iris scan, facial recognition) to strengthen the robustness of the authentication procedure. The combination of all three authentications is generally known as a three-factor authentication. In particular, biometric authentication has gained increasing importance during the last decade with the advent of smartphones and wearables. In addition, several studies have reported the feasibility of using wearables to authenticate users’ identity from the continuous collection of user-centric data, such as heart rate, body temperature, ECG signals or body motion [413,414,415]. Concerning device authentication, which cannot implement traditional protocols due to their energy and time consumption, a number of lightweight authentication protocols have already been presented [416,417,418,419].

##### Access Control Mechanisms

Access control limits the access of users or devices (i.e., subjects) to the resources (i.e., objects) of the system, by establishing a subject-to-object segregation [420]. For instance, patients may only access to their own information, physicians may only have access to the medical information of their patients, whereas nodes may only have access to the services associated to their own task. With scalability and flexibility in mind, numerous fine-grained access policies have been defined to enforce different access privileges to the system’s subjects. Extensively used, role-based access control (RBAC) models associate each subject to a role, and each role has a set of access permissions. Thus, a subject has as many permissions as the role indicates. This scheme combines both security and privacy to the system’s objects with usability and flexibility at the time to define the privileges and roles [421]. Alternatively, cryptography can also help define access control mechanisms in a more secure way with the attribute-based encryption (ABE)-based fine-grained access control. In this context, the information is encrypted with a set of attributes (e.g., department, age, gender…), and only the users fulfilling those attributes are able to decrypt the information and hence, gain access [422]. Furthermore, the work in [423] proposed the use of blockchain technology to decentralise the access control within an IoT environment with a privacy-preserving component.

##### Intrusion Detection Systems

The deployment of preventive mechanisms, such as intrusion detection systems (IDS), can help detect attacks at an early stage. IDS monitor and analyse the activities happening within a system, and alert once detecting unknown or potentially malicious activities. Depending on the deployment of the IDS, they can be network-based (NIDS), i.e., monitor data packets across the network for malicious activities, or host-based (HIDS), i.e., monitor all the activities occurring within an end device, such as the modification of files, operating system calls, running processes and the utilisation of resources. IDS are a mature technology in traditional environments, however, they might not yet be adequate in context-aware environments. In this context, IDS must be as lightweight as possible to minimise the overhead introduced in the system’s infrastructure, so that they do not interfere or significantly impact with the proper functioning of the system [424].

The most popular IDS method is based on anomaly detection. By properly defining the normal behaviour of the system (captured during a training phase), the real-time activity can be compared against the normal behaviour. When the distance between the normal and the real-time behaviour exceeds a predefined threshold, an alarm is raised. Although this method allows the detection of new kinds of attacks and malware, it is susceptible to high false alarm rates as the accuracy of the method depends on the behaviour captured during the training phase, which may be incomplete in some cases [425]. Another method is based on signatures, referring to the effects and patterns suffered by a system due to an attack. Storing all the signatures of known attacks in a database, IDS are able to detect whether the real-time behaviour of the system matches with any stored signature. Despite the high accuracy of this method, unknown attacks remain undetectable [426]. With the aim of combining the advantages of these two approaches, hybrid specification-based IDS methods have emerged [427].

##### Traceability of Digital Evidence

When systems are compromised, specific information about the attacks might remain in the systems, unless attackers have been able to completely destroy their footprint. Preserving the traceability of these digital evidence is paramount to report the incident to the judicial authorities and initiate an investigation to prosecute the criminals. However, this represents a challenge for judiciary forces that should be able to face these criminals’ behaviours in an efficient way and, in some case, from an international perspective. The procedure needed to obtain digital evidence, along with their recognition in a court of justice, should follow a standardised procedure accepted by most jurisdictions. This procedure should guarantee the origin of the evidence and the integrity of the chain of custody. As such, the lack of standardisation in the process of sharing and handling digital evidence among jurisdictions entails several disparities on how forensic reports are presented. Therefore, standardising the process used in the preparation of digital forensic reports is a crucial step towards producing high-quality reports and a way to facilitate the sharing and admissibility of reports across jurisdictions [428].

Despite the complexity of implementing similar mechanisms for the management and presentation of digital evidence in different jurisdictions, a series of international standards have been provided by ISO/IEC institutions with the aim to properly manage potential digital evidence from its collection to its reporting. For example, the ISO/IEC 27037 provides guidance with respect to the identification, collection, acquisition and preservation of digital evidence from different devices, such as storage media and mobile phones, among others [429]. In line with this standard, the ISO/IEC 27041 provides guidance to assure that the performed investigative process has been properly tested and meets the requirements of the investigation [430]; the ISO/IEC 27042 describes the correct conduct for analysis and interpretation of potential digital evidence to allow the correct evaluation, interpretation and reporting of the potential digital evidence [431]; and the ISO/IEC 27043 provides an overview of the investigation principles from the incident identification to its closure [432]. The correct implementation of these standards may be helpful to build trust and therefore, foster cross-border cooperation [433].

#### 4.3.4. Data Protection

In the management of personal information, there are a number of privacy concerns which arise throughout the entire data life cycle: from the storage and transmission over the network to their analysis and exploitation for secondary use. To prevent data misuse scandals and mitigate the impact of data leakages, legal privacy regulations, such as GDPR in Europe [434] and HIPAA in the United States [435], lay the foundations for the proper management and processing of personal information in the digital universe. Particularly, the enforcement of GDPR has strengthened people’s privacy rights and forced organisations to adopt privacy-by-design principles, including data minimisation, transparent and lawful processing, accountability and pseudonymisation or encryption. As medical and biometric data are categorised as highly sensitive data, the privacy issues are even more apparent, and smart healthcare systems must carefully implement all the proper privacy-preserving safeguards to ensure both data confidentiality and privacy [436]. In this section, some privacy-preserving models are introduced, and the usefulness of awareness programmes are outlined.

##### Privacy Protection Models

From a privacy perspective, the main objective was to break the link between personally identifiable information (e.g., ID number, full name, social security number…) with its corresponding confidential information (e.g., physiological parameters, biometric data…). Therefore, in the case of a data leakage, people’s identities cannot be seamlessly associated to confidential information. Pseudonymisation, a GDPR-friendly practice, can reduce privacy risks by masking individuals’ identities with artificial identifiers, called pseudonyms [437]. This strategy has to be considered when communicating medical data between two entities among the network, so that only the legitimate entities can correlate the pseudonym with its identity. In the case of eavesdropping, attackers are not able to identify the source belonging to the captured sensitive data.

Once information is stored within the HIS for secondary use or statistical purposes, data should undergo an anonymisation process ensuring that a third-party is not able to re-identify the individual’s identity from the data stored. In this context, in addition to the removal of personally identifiable information, a number of techniques can be applied to the data, such as noise addition (e.g., the physiological values are slightly different regarding the original ones) or micro-aggregation (e.g., creating groups of similar values and conserving only their centroids) [438]. In this aim, several privacy protection models have been proposed within the privacy protection literature, namely *k*-anonymity, *l*-diversity, *t*-closeness or differential privacy [439,440]. It is worth noting that the anonymisation procedure implies a trade-off between data quality and privacy: the more privacy, the lower the data quality.

##### Awareness Programmes

Organisations and public administrations should foster awareness programmes on cybersecurity and data protection to educate non-expert users. These programmes must provide high-quality updated information, tips, recommendations and campaigns that users could easily apply in their daily routines to prevent or mitigate user-oriented attacks. Among others, these programmes could be oriented for phishing, home/work computer security, mobile device security, secure remote working, best practices on strong passwords or Wi-Fi security. Unfortunately, these actions are often not applied in most organisations and when conducted, they are considered from a very generic perspective. Thus, in an intent to clarify the relationship between the human factor in phishing victimisation, the most recent research has been exploring the role of psychological traits and users’ susceptibility to phishing attacks. The results of this research will facilitate the creation of more effective awareness campaigns and therefore, contribute to protect people, companies and infrastructures [441].

## 5. Future Challenges and Research Opportunities

Despite the progressive adoption of context-aware environments, smart healthcare applications and services are still constrained to very specific scenarios and cannot exploit all their potential. Thanks to the latest developments in the manufacturing of IoT and high-speed communications networks, the implementation of these environments will certainly accelerate in the years to come, and when they are a reality, the ecosystem of smart healthcare will reach a higher dimension.

Consumer electronics, by means of wearables, IoT and IoMT devices, have been the linchpin of most health-oriented services to enhance one’s quality of life in the last decade. Significant advancements in the miniaturisation of sensors have opened the door to nanotechnology, which can revolutionise myriad aspects of healthcare and open the door to new frontiers and research opportunities, including disease diagnostics and monitoring, surgical devices, drug delivery and vaccine development. Although initial nanotechnology-based devices have already been set in the form of ingestible sensors and textile-based wearables, their use is still not generalised yet due to their costly manufacturing. Next-generation nanotechnology-based devices may consider smart pills with sensing, imaging and drug delivery capabilities for nanomedicine purposes, nanobots working as miniature surgeons with repairing capabilities of cellular structures, and nanofibres for regenerative medicine [442,443,444]. For instance, nanotechnology could play a key role in the fight against COVID-19 [445]. The optimism regarding nanotechnology has already enabled coining the term of the Internet of Nano-Things (IoNT) [446], whose success in the smart healthcare domain will mostly depend on the success to address its security concerns, not only regarding the safety of human lives, but also from the technological side. The development of security countermeasures in such technologies will certainly be a major technical challenge.

The increase in mobile devices integrating wireless communication capabilities adds complexity to the already challenging electromagnetic spectrum of context-aware environments. In these scenarios, the placement of the different devices from WSNs and WBANs can determine the correct performance of the entire system. Inadequate configurations can dramatically decrease the quality of service of devices operating in context-aware environments, leading to severe consequences in sensitive contexts, such as smart hospitals or smart ICUs. However, the continuous movement of both humans and wireless devices in these settings hinders the analysis of such communications systems. To this aim, radio-planning analyses in terms of coverage/capacity relations, power distribution, potential interference, power delay profiles and delay spread should be considered. Deterministic simulations based on ray optics, such as ray launching or ray tracing, are popular methods offering a reasonable trade-off between precision and computational cost. Many wireless technologies, including ZigBee, BLE and Bluetooth, have been studied in complex and highly dense environments such as hospitals and ICUs [447,448]. These analyses might not only help evaluate appropriate configurations of context-aware environments, but also anticipate and protect networks from malicious interference-based attacks, such as jamming attacks. Another inevitable challenge of next-generation communication networks is their evolution towards green communications. With the aim of reducing the alarming carbon footprint of current technologies due to their intensive demand, decreasing the energy consumption of communications is currently in the spotlight [449]. A number of low-power and green communication technologies are expected to gain popularity in the near future, including LPWAN and 5G models, which have already been evaluated under this model [450,451], as well as the 6G networks of tomorrow [452]. Energy-efficient lightweight security solutions will hence be a mandatory requirement in the wireless communication technologies of the future.

There are multiple emerging architectures that can enhance the security robustness of most current systems. However, their adoption in the smart healthcare domain is still at an embryonic stage. On the one hand, zero trust architectures [453] can be seen as an interesting solution to enhance security in systems. These architectures offer a security model based on the premise of not trusting any entity until a validation, legitimation and authorisation process has been passed. The model supports the implementation of least privileged access and continually requires the identification of actors who have gained access to the network. Although the adoption of zero trust models is not trivial [454], its application in smart healthcare applications might provide several benefits for users and bring new levels of security. On the other hand, the use of blockchain technology could provide a new model in the smart healthcare industry, by making electronic health records more efficient, transparent and secure. Thus, to secure information, smart contracts can be seen as an interesting alternative to existing systems as they remove the need for a mediator. In the near future, this technology could allow organisations creating a secure system to store patient records and therefore enabling faster diagnosis and interventions to each patient. However, despite the numerous opportunities that blockchain could provide in the smart healthcare sector, it also raises several challenges that should be addressed in future research. In this line, [455] highlighted the need to address issues such as patient data interoperability, secure storage in Cloud systems and data control in blockchain, among others.

Equally impressive is the evolution of artificial intelligence (AI) in the last decade, particularly that machine learning and deep learning. The maturity of this field enables influencing other areas. Today, with fast-evolving security threats and attacks, AI-based applications for cybersecurity offer a strategic advantage to thwart malicious endeavours of attackers at contained costs [456]. Many systems, including HIS and other components involved in smart healthcare environments, can enhance their robustness, response and resilience through AI [457]. For example, security attacks could be mitigated or defeated autonomously, including zero-day attacks whose value in the black market would decrease, security countermeasures could be launched on-the-fly according to attack severity, and honeypots could be dynamically generated. Moreover, AI can also fuel novel cybersecurity countermeasures to enable the detection of sophisticated malware and phishing, and the development of advanced IDS with excellent accuracy rates [458]. Recently introduced, multi-agent systems are expected to be promising solutions to face security threats in distributed architectures [459,460]. Although AI can significantly improve security solutions, it is a double-edged sword because it could also facilitate novel sorts of attacks that adversaries might exploit to generate new categories of vulnerabilities and unforeseen security threats may arise. With the aim of making AI reliable for cybersecurity, some development and monitoring practices should be followed [457], and ethical and legal challenges must be properly addressed [461].

Technology has irreversibly transformed healthcare systems and it is apparent to expect that it will still reshape them even more in the future. Future developments of healthcare systems will be partly influenced by the context where these health services will be provided. Hence, since smart healthcare was founded under the concept of smart cities and context-aware environments, next-generation healthcare paradigms might be founded under the contexts and environments of tomorrow. In particular, the conceptualisation of connected learning theories, such as connectivism [462], along with recent technological shifts, such as IoT, ubiquitous computing, IA and big data, have allowed the development of cognitive cities, a novel augmented urban paradigm that is gathering the attention of the research community. Cognitive cities [463], a particular implementation of a cognitive system at a very large scale, are able to learn, adapt their behaviour based on past experiences, and sense, understand and respond to changes in their immediate environment. Note that cognitive cities augment smart cities with learning and behavioural capabilities to face the challenges of future mega-cities. In this specific context, one might find appropriate coining a specific healthcare paradigm for cognitive environments: cognitive healthcare. To make cognitive healthcare a reality, numerous challenges will need to be addressed, including security and privacy issues inherited from the very cognitive environments [464]. Although cognitive healthcare is still ahead in the future, setting the ground for potential security and privacy problems can be achieved at present.

## 6. Conclusions

This article has addressed the deployment of effective and secure smart healthcare services in context-aware environments from a technical perspective. More specifically, we addressed the issue facing devices in the sensing layer, able to collect both user-centric attributes, such as cardiovascular activity, respiratory rate or location, and contextual attributes, such as air temperature or air pollution. These devices must be met with adequate accuracy, size, cost and power consumption to be suitable for smart health scenarios. As a result, all data can be conveyed, either using specific sensors networks, namely WBANs or WSNs, to the healthcare information systems that provide the corresponding services. Likewise, with the aim of enabling physicians, medical staff and automated processes to analyse these data and provide real-time diagnosis, suggest personalised treatments and raise alarms to emergency services in specific situations, this study provides a throughout description of a large number of wireless technologies. In particular, we observed that Bluetooth/BLE is a prominent technology in WBANs, and ZigBee can be used in such networks as well as in wider sensor networks. Promising technologies including 5G cellular networks are paving the way for scalable architectures for the transmission of large volumes of data under real-time constraints.

Moreover, we also addressed the issue of information security in the smart healthcare context, which is paramount due to the high sensitivity of the information handled. Indeed, sensor networks are prone to attacks and if no specific measures are considered, smart healthcare services are doomed to fail. To address this aspect, we anatomised and classified the information security requirements, attacks and solutions in smart healthcare systems. Attacks against sensing devices, communications, information systems and users were also detailed. In order to provide the whole system with security properties, the capabilities of the constrained resources and the networks scalability must be considered. Hence, proposals such as lightweight cryptography, DDoS countermeasures and authentication protocols are bound to be the basis of security protocols in smart healthcare scenarios. Moreover, since the management of personal information arises a number of privacy concerns, we also recalled the basics of data protection, either from a technical perspective using privacy models and from a non-technical perspective with educational awareness programmes.

In a nutshell, in this article, we described a wide range of technologies and protocols, and we demonstrated that there exist a number of alternatives to be considered when designing and deploying smart healthcare services. However, we omitted some other aspects that could also be of interest. For instance, the interoperability problem related to the complex ecosystem of protocols, standards and manufacturers, specifically in sensor devices. Furthermore, education on the right use of technology, awareness programmes about cybersecurity and data privacy, ethical aspects of smart healthcare and their corresponding legal initiatives are only in their very early stages.

## Figures and Tables

**Table 1:** Taxonomy of the attributes to be considered for smart health.

User-Centric		Contextual	
Heart rate	Blood oxygen	Air temperature	Air humidity
Blood pressure	Blood glucose	Barometric pressure	Air pollution
Body temperature	Skin temperature	Water contamination	Acoustic contamination
Respiratory rate	Brain activity	Electromagnetic radiation	Seismic activity
Muscular activity	Electrodermal activity		
Hydration	Location		
Body motion			

**Table 2:** Characteristics of the methods for cardiovascular activity sensing.

Attribute	Method	Sensor	Device/ Wearable	Location	Properties	Suitable for Smart Health
Heart rate	Traditional ECG	Skin electrodes	Holter monitor	Chest	✓ Accuracy ~ Cost ~ Cont. monit. ✕ Non invasive	✕
Heart rate	Wireless ECG	Skin electrodes	Patch Band Textile	Chest Arm	✓ Accuracy ~ Cost ✓ Cont. monit. ~ Non-invasive	✓
Heart rate	PPG	Pulse oximeter	Smartwatch Wristband Ring	Wrist Finger	~ Accuracy ✓ Cost ✓ Cont. monit. ✓ Non-invasive	✓
Heart rate	BCG	Tilt Force Pressure	Patch	Chest	✕Accuracy ~ Cost ~ Cont. monit. ✓ Non-invasive	~
Heart rate	PCG	Sound	Microphone Smartphone Electronic stethoscope	Chest	✕ Accuracy ✓ Cost ~ Cont. monit. ✓ Non-invasive	~
Blood oxygen	ABG	Chemical	Chemical analyser	Arm Wirst	✓ Accuracy ✕ Cost ✕ Cont. monit. ✕ Non-invasive	✕
Blood oxygen	PPG	Pulse oximeter	Smartwatch Strap Band Textile	Wrist Earlobe Finger	~ Accuracy ✓ Cost ✓ Cont. monit. ✓ Non-invasive	✓
Blood pressure	Traditional	Pressure	Sphygmomanometer	Arm	✓ Accuracy ✓ Cost ✕ Cont. monit. ✕ Non-invasive	✕
Blood pressure	PTT (ECG and PPG)	Pulse oximeter Electrodes	Smartwatch Band Patch	Wrist Arm Ear Chest	✓ Accuracy ~ Cost ✓ Cont. monit. ✓ Non-invasive	✓
Blood glucose	Traditional (chemical)	Electrochemical	Glucose meter	Finger	✓ Accuracy ✓ Cost ✕ Cont. monit. ✕ Non-invasive	✕
Blood glucose	Epidermal chemical	Electrochemical	Wristband Patch Tattoo	Wrist Arm	~ Accuracy ~ Cost ✓ Cont. monit. ✓ Non-invasive	✓
Blood glucose	Optical spectroscopy	Photo-sensor Infrared	Wristband Patch	Wrist Finger Earlobe	~ Accuracy ~ Cost ✓ Cont. monit. ✓ Non-invasive	✓

**Table 3:** Characteristics of the methods for temperature sensing.

Attribute	Method	Sensor	Device/ Wearable	Location	Properties	Suitable for Smart Health
Body temperature	Traditional (chemical)	Mercury	Mercury-in- glass thermometer	Oral Rectal	✓ Accuracy ✓ Cost ✕ Cont. monit. ✕ Non-invasive	✕
Skin temperature	Electrical	Thermistor	Patch Band	Arm Chest Ear Forehead	~ Accuracy ✓ Cost ✓ Cont. monit. ✓ Non-invasive	✓
Skin temperature	Electrical	Thermocouple	Patch Band	Arm Chest Ear Forehead	✕ Accuracy ✓ Cost ✓ Cont. monit. ✓ Non-invasive	~
Skin temperature	Optical	FBG Infrared	Smartwatch Band Patch Textile	Wrist Chest Ear Forehead	~ Accuracy ✓ Cost ✓ Cont. monit. ✓ Non-invasive	✓

**Table 4:** Characteristics of the methods for respiratory rate sensing.

Attribute	Method	Sensor	Device/ Wearable	Location	Properties	Suitable for Smart Health
Respiratory rate	Traditional (observation of chest or abdomen)	-	-	-	✓ Accuracy ✓ Cost ✕ Cont. monit. ✓ Non-invasive	✕
Respiratory rate	Chest wall strain	Resistive Capacitive Inductive	Patch Belt Textile	Chest	✓ Accuracy ✓ Cost ✓ Cont. monit. ✓ Non-invasive	✓
Respiratory rate	Electrical impedance	Impedance	Patch Belt Textile	Chest	✓ Accuracy ✓ Cost ✓ Cont. monit. ✓ Non-invasive	✓
Respiratory rate	Motion (contact)	IMU	Patch Belt Textile	Chest Abdomen	✓ Accuracy ✓ Cost ✓ Cont. monit. ✓ Non-invasive	✓
Respiratory rate	Acoustic	Microphone	Microphone Headset	Nose Mouth Chest	✕ Accuracy ✓ Cost ✓ Cont. monit. ~ Non-invasive	~
Respiratory rate	Air temp. (electrical)	Thermistor Thermocouple Pyroelectric	Headset Patch	Nose Mouth	~ Accuracy ✓ Cost ✓ Cont. monit. ~ Non-invasive	~
Respiratory rate	Air humid. (electrical)	Capacitive Resistive Nanocrystal	Headset Patch	Nose Mouth	~ Accuracy ✓ Cost ✓ Cont. monit. ~ Non-invasive	~
Respiratory rate	Cardiac act. modulation	Pulse oximeter Electrodes	Smartwatch Band Patch	Wrist Chest	✓ Accuracy ~ Cost ✓ Cont. monit. ✓ Non-invasive	✓
Respiratory rate	Motion (contactless)	Camera	RGB camera Smartphone	-	~ Accuracy ✓ Cost ✕ Cont. monit. ✓ Non-invasive	~
Respiratory rate	Thermal imaging	Camera	Infrared camera	-	~ Accuracy ✕ Cost ✕ Cont. monit. ✓ Non-invasive	~
Respiratory rate	Ultrasonic	Ultrasonic prox. Capacitive	Recording device	-	~ Accuracy ✕ Cost ✕ Cont. monit. ✓ Non-invasive	~

**Table 5:** Characteristics of the methods for brain activity sensing and muscular activity sensing.

Attribute	Method	Sensor	Device/ Wearable	Location	Properties	Suitable for Smart Health
Brain activity	Traditional EEG	Skin electrodes	Head cap	Scalp	✓ Accuracy ✕ Cost ✕ Cont. monit. ✕ Non-invasive	✕
Brain activity	Wireless EEG	Skin electrodes	Headband Headset Tattoo	Scalp Head Forehead Ear	~ Accuracy ~ Cost ✓ Cont. monit. ✓ Non-invasive	✓
Brain activity	fNIRS	Optodes	Head cap	Scalp Head	✓ Accuracy ~ Cost ~ Cont. monit. ~ Non-invasive	✕
Brain activity	MEG	Optically pumped magnetometeres	Head cap	Scalp Head	✓ Accuracy ✕ Cost ~ Cont. monit. ~ Non-invasive	✕
Brain activity	PET	Photosensor Photodiode	Head cap Helmet	Head	✓ Accuracy ✕ Cost ✕ Cont. monit. ✕ Non-invasive	✕
Muscular activity	Intramuscular EMG	Monopolar or concentric electrodes	Needle and recording device	Region of interest	✓ Accuracy ✕ Cost ✕ Cont. monit. ✕ Non-invasive	✕
Muscular activity	Surface EMG	Skin electrodes	Patch Band Cap Textile	Region of interest	~ Accuracy ✓ Cost ✓ Cont. monit. ✓ Non-invasive	✓
Muscular activity	MMG	Accelerometer Pressure Force-sensitive	Patch Band	Region of interest	~ Accuracy ✓ Cost ✓ Cont. monit. ✓ Non-invasive	✓

**Table 6:** Characteristics of the methods for electrodermal activity sensing and hydration sensing.

Attribute	Method	Sensor	Device/ Wearable	Location	Properties	Suitable for Smart Health
Electrodermal activity	Electrical	Skin electrodes (wired)	Smartwatch Band Strap	Wrist Finger	✓ Accuracy ✓ Cost ✓ Cont. monit. ~ Non-Invasive	∼
Electrodermal activity	Electrical	Skin electrodes (wireless)	Smartwatch Band Strap	Wrist Finger	✓ Accuracy ✓ Cost ✓ Cont. monit. ✓ Non-invasive	✓
Hydration	Traditional (observation of eyes or lips)	-	-	-	✓ Accuracy ✓ Cost ✕ Cont. monit. ✓ Non-invasive	✕
Hydration	Optical spectroscopy	Infrared	Band Patch Textile	Wrist Arm Head	✓ Accuracy ~ Cost ~ Cont. monit. ✓ Non-invasive	✓
Hydration	Electromagnetic	Impedance Capacitive	Band Patch Textile	Wrist	~ Accuracy ✓ Cost ~ Cont. monit. ✓ Non-invasive	✓
Hydration	Epidermal chemical	Electrochemical	Band Patch Tattoo Textile	Wrist Arm	✓ Accuracy ~ Cost ~ Cont. monit. ✓ Non-invasive	✓

**Table 7:** Characteristics of the methods for location sensing and body motion sensing.

Attribute	Method	Sensor	Device/ Wearable	Location	Properties	Suitable for Smart Health
Location	Satellite (outdoor)	GPS GLONASS Galileo	Smartphone Smartwatch Band	Any	✓ Accuracy ✓ Cost ✓ Cont. monit. ✓ Non-invasive	✓
Location	Proximity (indoor)	BLE beacon WPS RFID UWB	IoT Access point Tag	-	✓ Accuracy ✓ Cost ~ Cont. monit. ✓ Non-invasive	✓
Body motion	Optical motion capture	Camera	Camera Marker	Markers distributed in the body	✓ Accuracy ✕ Cost ✕ Cont. monit. ✕ Non-invasive	✕
Body motion	Optical	Camera	RGB-depth camera	-	~ Accuracy ~ Cost ~ Cont. monit. ✓ Non-invasive	~
Body motion	Kinematic	IMU	Band Patch Textile	Region of interest	~ Accuracy ✓ Cost ✓ Cont. monit. ✓ Non-invasive	✓

**Table 8:** Characteristics of the methods for air temperature sensing, air humidity sensing and barometric pressure sensing.

Attribute	Method	Sensor/Device	Properties	Suitable for Smart Health
Air temperature	Electrical	Thermocouple	✕ Accuracy ✓ Cost ✓ Response time ✗ Energy consumption	~
Air temperature	Electrical	Resistance temperature detector	✓ Accuracy ~ Cost ✕ Response time ✓ Energy consumption	~
Air temperature	Electrical	Thermistor	✓ Accuracy ~ Cost ✓ Response time ✓ Energy consumption	✓
Air temperature	Electrical	Semiconductor integrated circuit	~ Accuracy ✓ Cost ✓ Response time ✓ Energy consumption	✓
Air humidity	Electrical	Capacitive	✓ Accuracy ✕ Cost ✓ Response time ✓ Energy consumption	~
Air humidity	Electrical	Resistive	~Accuracy ✓ Cost ~ Response time ✓ Energy consumption	✓
Air humidity	Optical	Fibre-optic	✓ Accuracy ✕ Cost ~ Response time ✓ Energy consumption	~
Barometric pressure	MEMS	Piezoresistive pressure	✓ Accuracy ✓ Cost ✓ Response time ✓ Energy consumption	✓

**Table 9:** Characteristics of the methods for air pollution sensing, water contamination sensing and acoustic contamination sensing.

Attribute	Method	Sensor/Device	Properties	Suitable for Smart Health
Air pollution	Optical spectroscopy	Infrared Fluorescence	✓ Accuracy ✕ Cost ✕ Response time ~ Energy consumption	✕
Air pollution	Chemiresistive	MOS	~ Accuracy ✓ Cost ✓ Response time ~ Energy consumption	✓
Air pollution	Electrochemical	Electrochemical	✓ Accuracy ✕ Cost ✓ Response time ✓ Energy consumption>	~
Water contamination	Traditional (chemical)	In-lab instrumentation	✓ Accuracy ✕ Cost ✕ Response time ✕ Energy consumption	✕
Water contamination	Electrochemical	Resistive Capacitive Conductance	~ Accuracy ~ Cost ✓ Response time ✓ Energy consumption	✓
Water contamination	Optical	CMOS camera	~ Accuracy ✓ Cost ✓ Response time ✓ Energy consumption	✓
Acoustic contamination	Acoustic	Microphone	✓ Accuracy ✓ Cost ✓ Response time ✓ Energy consumption	✓

**Table 10:** Characteristics of the methods for electromagnetic radiation sensing and seismic activity sensing.

Attribute	Method	Sensor/Device	Properties	Suitable for Smart Health
Electromagnetic radiation	Electrical	Geiger–Müller tubes	✓ Accuracy ✕ Cost ~ Response time ~ Energy consumption	~
Electromagnetic radiation	Optical	Fibre-optic	✓ Accuracy ~ Cost ✓ Response time ~ Energy consumption	✓
Seismic activity	Traditional (motion)	Seismometer	✓ Accuracy ✕ Cost ~ Response time ~ Energy consumption	✕
Seismic activity	Kinematic	Accelerometer	~ Accuracy ✓ Cost ~ Response time ✓ Energy consumption	✓
Seismic activity	Optical	Opto-mechanical	~ Accuracy ~ Cost ~ Response time ✓ Energy consumption	✓

**Table 11:** Comparison of the main wireless communication technologies for smart healthcare (I).

	Bluetooth	BLE	ZigBee	IEEE 802.15.6	Wi-Fi
**Frequency** **bands**	2.4 GHz	2.4 GHz	868/915 MHz 2.4 GHz	14–29 MHz (HBC) 400–2400 MHz (NB) 3.2–10.3 GHz (UWB)	2.4/5 GHz
**Radio coverage**	Short/medium	Medium	Short/medium	Short	Medium
	10–100 m	400 m	10–100 m	2 m	50–100 m
**Data rate**	Moderate	Moderate	Low	Low/moderate	High
	1–3 Mbps	1–2 Mbps	20–250 kbps	10 kbps–15 Mbps	400 Mbps–10 Gbps
**Latency**	Moderate	Very low	Very low/Low	Low/moderate	Low
	100 ms	10 ms	10–30 ms	125 ms	50 ms
**Power**	Moderate	Very low	Very low/low	Very low	High
	0.2–0.5 W	10 mW	1–60 mW	0.1–3 mW	0.8–1 W
**Size**	8	32,000	65,000	256	250
**Topology**	Scatternet	Star, mesh	Star, tree, mesh	Star, multi-hop	Star, mesh, ad hoc
**Security**	56,64,128-bit AES	128-bit AES	128-bit AES	Level 1/Level 2	128,256-bit AES
**Cost**	Medium	Low	Low	Low	High
**WBAN tier**	Tier 1/Tier 2	Tier 1/Tier 2	Tier 2	Tier 1	Tier 2/Tier 3
**Suitable for** **smart health**	~	✓	~	✓	✓

**Table 12:** Comparison of the main wireless communication technologies for smart healthcare (and II).

	4G/LTE	5G	LoRa	SigFox	NB-IoT
**Frequency** **bands**	0.7–2.6 GHz	600–700 MHz 2.5–3.8 GHz 25–100 GHz	863–928 MHz	868/915 MHz	800–900 MHz
**Radio coverage**	High	Medium/high	High	High	High
	10 km	300 m–1 km	5–20 km	10–50 km	15 km
**Data rate**	High	Very high	Very low	Very low	Low
	10–300 Mbps	1–20 Gbps	37.5 kbps	100–600 bps	250 kbps
**Latency**	Low	Very low	High	High	High
	50–70 ms	1–10 ms	3 s	10 s	1 s
**Power**	Moderate	Low	Low	Low	Low
	250–700 mW	N/A	25 mW	10–100 mW	20–200 mW
**Size**	Thousands per km^2^	1 million per ${\mathrm{km}}^{2}$	1000	1,000,000	50,000
**Topology**	Cellular	Cellular	Star of stars	Star	Star
**Security**	128-bit	256-bit	128-bit AES	Optional	128,256-bit
**Cost**	Medium	High	Low	Low	Low
**WBAN tier**	Tier 2/Tier 3	Tier 2/Tier 3	Tier 2	Tier 2	Tier 2
**Suitable for** **smart health**	✓	✓	~	✕	~

**Table 13:** Summary and classification of security attacks in smart healthcare systems.

Attack	Target Actor	Nature	Origin	Launch Method	TCP/IP Layer	Requirements Threats
Node capture	Nodes	Active	External	Physical	Network interface	Confidentiality Non-repudiation Authentication Privacy
False data injection	Nodes	Active	Internal	Physical	Network interface	Integrity
Sleep deprivation	Nodes	Active	External	Physical Logical	Network interface	Availability
Side-channel	Nodes	Passive Active	External	Side-channel	Network interface	Confidentiality Availability
Firmware update	Nodes	Active	External	Logical	Network interface	Confidentiality Non-repudiation Authentication Authorisation
Eavesdropping	Communications	Passive	External	Logical	Network interface Network	Confidentiality Privacy
Data tampering	Communications	Active	Internal	Physical	Network interface	Integrity
Replay	Communications	Active	Internal	Physical	Network	Integrity Authentication Authorisation
Spoofing	Communications	Active	Internal External	Physical	Network interface Network Transport Application	Integrity
Man-in-the-middle	Communications	Active	Internal External	Logical	Network Transport	Confidentiality Integrity Authentication Privacy
Flooding	Communications	Active	Internal External	Logical	Network Transport Application	Availability
Jamming	Communications	Active	External	Physical	Network interface	Availability
Black hole	Communications	Active	Internal	Physical	Network	Availability
Malware	HIS Nodes	Active	External	Logical	Application	Confidentiality Integrity Availability Non-repudiation Authentication Authorisation Privacy
Data leakage	HIS	Passive	External	Logical	Application	Confidentiality Privacy
Phishing	Users	Active	External	Logical	Application	Confidentiality Authentication Authorisation Privacy

**Table 14:** Summary and classification of security solutions in smart healthcare systems.

Type	Solution	Actor	TCP/IP Layer	Requirements Protected
Secure communications	Lightweight cryptography	Nodes Communications HIS	Network interface	Confidentiality Integrity Non-repudiation Authentication
	Key management	Nodes HIS	Network interface	Confidentiality Authentication
Always-on systems	Secure routing	Communications	Network	Availability
	DDoS countermeasures	Nodes Communications HIS	Network	Availability
Trust management	Authentication protocols	Nodes HIS	Transport Application	Authentication Confidentiality Privacy
	Access control mechanisms	HIS	Application	Authentication Confidentiality Privacy
	Intrusion detection systems	Communications HIS	Network Transport Application	Confidentiality Integrity Availability Authentication Privacy
	Traceability of digital evidence	HIS	Application	Integrity
Data protection	Privacy protection models	HIS	Application	Privacy
	Awareness programmes	Users	-	Privacy

## Abbreviations

The following abbreviations are used in this manuscript:

ABE	Attribute-Based Encryption
ABG	Arterial Blood Gas
AES	Advanced Encryption Standard
AI	Artificial Intelligence
ARP	Address Resolution Protocol
BCG	Ballistocardiography
BLE	Bluetooth Low Energy
COPD	Chronic Obstructive Pulmonary Disease
DDoS	Distributed Denial of Service
DNS	Domain Name System
DoS	Denial of Service
ECC	Elliptic-Curve Cryptography
ECG	Electrocardiography
EDA	Electrodermal Activity
EEG	Electroencephalography
EMG	Electromyography
FBG	Fibre Bragg Grating
fNIRS	Functional Near-Infrared Spectroscopy
GDPR	General Data Protection Regulation
GPS	Global Positioning System
HBC	Human Body Communication
HIDS	Host-Based Intrusion Detection Systems
HIPAA	Health Insurance Portability and Accountability Act
HIS	Healthcare Information System
HTTP	Hypertext Transfer Protocol
ICMP	Internet Control Message Protocol
ICT	Information and Communication Technologies
ICU	Intensive Care Unit
IDS	Intrusion Detection Systems
IEC	International Electrotechnical Commission
IMU	Inertial Measurement Units
IoMT	Internet of Medical Things
IoT	Internet of Things
IP	Internet Protocol
ISO	International Organisation for Standardisation
LPWAN	Low-Power Wide-Area Networks
LTE	Long-Term Evolution
MAC (address)	Media Access Control
MAC (code)	Message Authentication Code
MEG	Magnetoencephalography
MEMS	Microelectromechanical Systems
MitM	Man-in-the-Middle
MMG	Mechanomyography
MOS	Metal Oxide Semiconductor
NB	Narrowband
NFC	Near-Field Communication
NIDS	Network-Based Intrusion Detection Systems
OSI	Open Systems Interconnection
OSINT	Open Source Intelligence
PCG	Phonocardiography
PET	Positron-Emission Tomography
PPG	Photoplethysmography
PTT	Pulse Transit Time
RBAC	Role-Based Access Control
RFID	Radio Frequency Identification
RGB	Red Green Blue
RSA	Rivest–Shamir–Adleman
RTS/CTS	Request to Send/Clear To Send
SHA	Secure Hash Algorithm
TCP	Transmission Control Protocol
UV	Ultraviolet
UWB	Ultra Wideband
WBAN	Wireless Body Area Networks
WPA	Wi-Fi Protected Access
WPS	WiFi-based Positioning System
WSN	Wireless Sensor Networks
